# Genotype-phenotype associations in familial exudative vitreoretinopathy: A systematic review and meta-analysis on more than 3200 individuals

**DOI:** 10.1371/journal.pone.0271326

**Published:** 2022-07-13

**Authors:** Xiaona Wang, Jun Chen, Hui Xiong, Xuhui Yu

**Affiliations:** Eye Hospital, The First affiliated Hospital of Harbin Medical University, Key Laboratory of Basic and Clinical Research of Heilongjiang Province, Harbin, Heilongjiang, China; Roskamp Institute, UNITED STATES

## Abstract

**Objective:**

To systematically review the relationship between genotypes and clinical phenotypes of Familial exudative vitreoretinopathy (FEVR) to support risk estimation and therapeutic decisions.

**Design:**

Systematic review with meta-analysis.

**Data sources:**

The data of our study were collected from PubMed, Embase, Web of Science, Cochrane, CBM, China National Knowledge Infrastructure (CNKI), WAN FANG and VIP databases since inception to August 2021.

**Results:**

A total of 3257 patients from 32 studies were included according to the inclusion and exclusion criteria. Among all the cases, the mutation frequencies of LRP5, FZD4, NDP, TSPAN12, ZNF408 and KIF11 were 13.6%, 11.5%, 4.6%, 6.7%, 1.6%, and 5.7%, respectively. We found that the patients with NDP and FZD4 suffer more severe symptoms, among which 86.4% patients of NDP and 78.6% patients of FZD4 were in the advanced stage of FEVR. Retinal detachment is the most frequent symptom with patients of LRP5 and NDP mutations, accounting for 51.9% and 64.5%, respectively. For the patients with the mutation of TSPAN12, retinal fold is the most common clinical manifestation, and suffer the mildest clinical phenotypes compared with the other three genes.

**Conclusion:**

The results of the meta-analysis indicate that different types of genetic mutations occur at different frequencies. In addition, the clinical manifestations of FEVR are related to the type of gene mutation. Therefore, targeted treatment strategies and follow-up recommendations should be adopted for different pathogenic genes of FEVR.

## Introduction

FEVR is recognized as a family hereditary disease that can cause blindness and irreversible damage to the retina. Early diagnosis and timely treatment are of great significance to prevent serious complications and improve the vision and prognosis of patients. A growing body of research is trying to develop more accurate early diagnosis methods by combining genetic diagnosis. Currently, a total of 6 types of pathogenic genes related to FEVR have been identified, including LRP5, FZD4, NDP, TSPAN12, ZNF408, and KIF11 [1]. The incidence of gene mutation is about 40%-50% [2–4]. The pathogenesis of FEVR is complex and its clinical manifestations are diverse [5]. Patients with mild disease showed only avascular areas and neovascularization around the retina, without any clinical symptoms. Among patients with severe disease, there will be fiber proliferation pulling macula and blood vessels, forming retinal folds, and even developing vitreous hemorrhage and retinal detachment, eventually leading to complete blindness [6].

Several studies [7, 8] have explored the relationship between genotypes and clinical phenotypes of FEVR to support risk estimation and therapeutic decisions. However, due to the low incidence of FEVR and large heterogeneity of clinical manifestations, these studies had very limited sample sizes and genotypes, leading to no statistical conclusions being drawn. In this paper, meta-analysis is adopted to study the frequency of gene mutation, disease staging, clinical manifestations and explore the relationship between genotype and clinical manifestations by integrating a large number of cases of FEVR. Our study aimed to draw statistical and reliable conclusions about the correlation between genotypes and clinical manifestations. Combining gene mutation diagnosis can promote more extensive follow-up, and provide more comprehensive prenatal diagnosis and genetic counseling for patients with a positive family history of pathogenic genes.

## Methods

### Literature search

The cases of FEVR involved in our study were obtained from the following databases: PubMed, Embase, Web of Science, Cochrane, CBM, China National Knowledge Infrastructure (CNKI), Wan Fang, and VIP.

Medical Subject Headings (MeSH), free words combined with familial exudative vitreoretinopathies, and genes were used as keywords to retrieve high-quality related studies in English or Chinese from 2000 to the present (S1 Table).

### Inclusion and exclusion criteria

Inclusive criteria: 1) The samples are from random or unselected populations. 2) Papers report the total number of FEVR probands with gene mutation cases and sample size, or the sample size could be calculated from the reported data. 3) The study reported patients with the above six gene mutations or included the clinical stage or specific clinical manifestations of patients with FEVR. 4) Articles reporting at least one patient with one gene mutation.

Exclusive criteria: 1) The studies only with one case. 2) Non-research articles such as review articles, editorials, and conference reports. 3) Papers about the mechanism of FEVR or molecular mechanism. 4) Documents with duplicate data or incomplete data. 4) Documents whose full text is not available.

### Data screening and assessment

Based on the tiles and abstracts, Xiaona Wang, Jun Chen and Hui Xiong performed the preliminary screening. Then, the full text was checked to determine the final qualified study. When more than one studies report the same cases, only the most comprehensive study was included to avoid the double-counting data.

Each patient with gene mutations was recorded by the highest stage of FEVR in either eye refer to the most commonly five-stage classification standard [9]. At the same time, we identified patients with mild or severe according to Trese’s Staging System [10], where Stage 1–2 was defined as mild FEVR and Stage 3–5 as severe FEVR. In clinical, mild patients often show no symptoms, while severe patients have a high probability of developing blindness due to severe retinal damage such as retinal detachment. All the research were evaluated by the cross-sectional study Quality Evaluation Scale recommended by the Agency for Health Research and Quality (AHRQ) [11]. The scale contains 11 items, with a total score of 11, among which 0–3 is low quality, 4–7 is medium quality, and 8–11 is high quality (S2 Table).

### Statistical analysis

95% CI and statistical heterogeneity were performed for each included study using the meta-analysis package in R which is widely used in the field of statistics. When the P-value is less than 0.1 or I^2^ >50%, heterogeneity is considered. In this case, the random influence model is adopted; Otherwise, a fixed model will be used.

## Results

**Fig 1:**
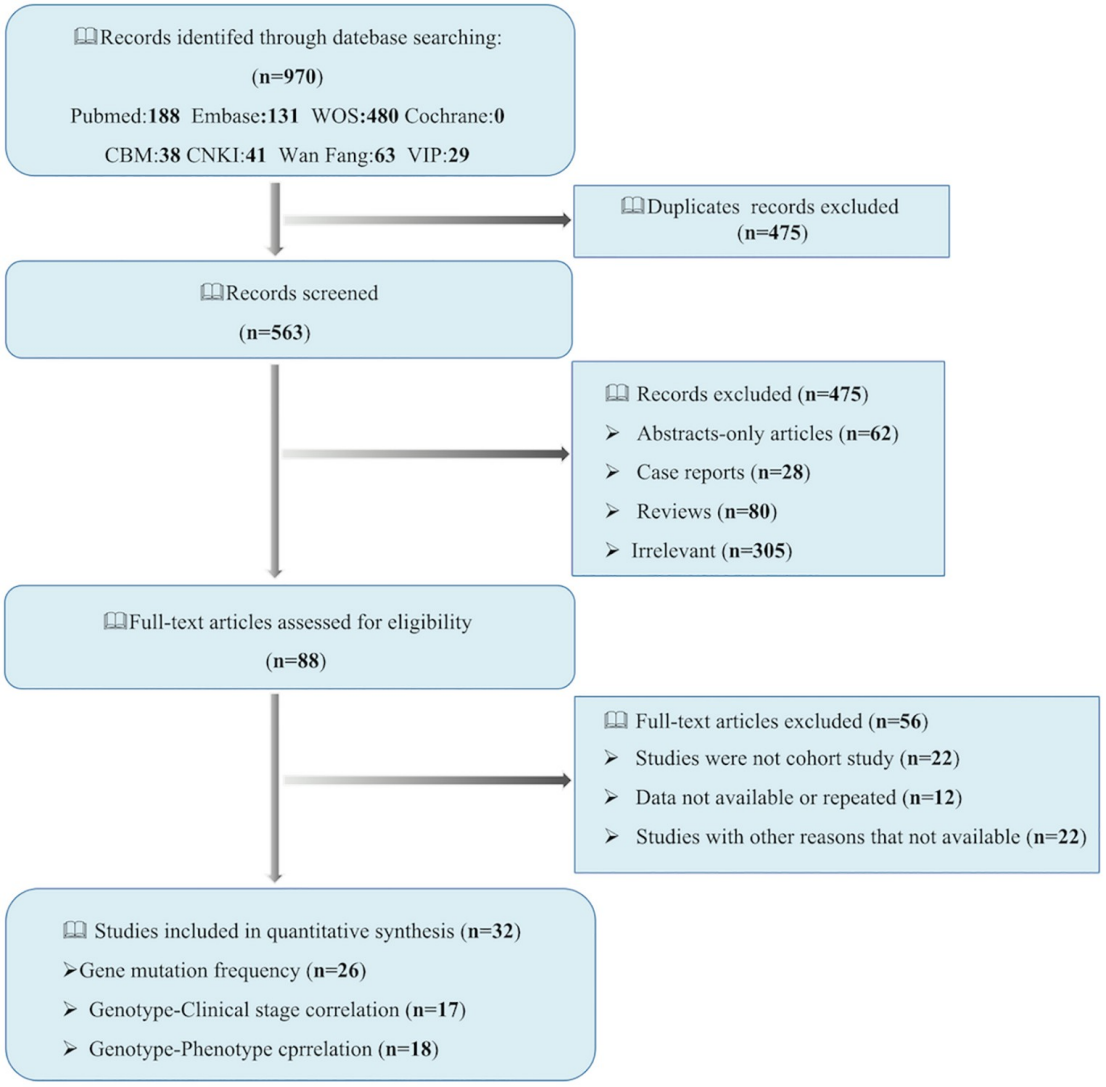
Flow diagram of the literature search process.

According to the keywords, a total of 970 articles were retrieved from the seven databases. 3257 FEVR patients were included from 32 studies and the search process was shown in Fig 1. Table 1 showed the detailed information and quality assessment of each study. Among them, 26 studies involved gene mutation frequency, and 17 studies focused on the correlation between gene mutation types and clinical stages. In addition, 18 studies reported the correlation between gene mutation types and clinical manifestations.

**Table 1:** Characteristics of the studies included in the meta-analysis.

Author	Year	country	Sample	Case	Genes screened	Scores
Fengqin Rao [12]	2017	China	31	12	LRP5, FZD4, NDP, TSPAN12, ZNF408, KIF11	9
Chunli Chen [7]	2020	China	722	349	LRP5, FZD4, NDP, TSPAN12, ZNF408, KIF11	9
Tian Tian [13]	2019	China	516	304	LRP5, FZD4, NDP, TSPAN12, ZNF408	10
Li, Jiakai [3]	2018	China	389	161	LRP5, FZD4, NDP, TSPAN12, ZNF408, KIF11	9
Li, Yian [14]	2016	China	130	64	LRP5, FZD4, NDP, TSPAN12, ZNF408	10
Chonglin Chen [2]	2020	China	62	30	LRP5, FZD4, NDP, TSPAN12, ZNF408, KIF11	9
Wen-min Sun [15]	2021	China	311	21	TSPAN12	8
Ganeswara Rao.Musada [16]	2016	India	110	27	FZD4, NDP, TSPAN12, ZNF408	9
Xiong Zhu [17]	2020	China	32	5	FZD4, NDP	9
Jason Salvo [18]	2015	America	92	45	LRP5, FZD4, NDP, TSPAN12, ZNF408	10
Soo Hyun Seo [19, 20]	2016	Korea	51	21	LRP5, FZD4, NDP, TSPAN12, ZNF408	10
Hiroyuki Kondo [21]	2003	Japan	24	5	FZD4	9
Hiroyuki Kondo [22]	2011	Japan	90	3	TSPAN12	9
Hiroyuki Kondo [23]	2007	Japan	62	4	NDP	9
Johane M Robitaille [24]	2011	Canada	68	12	FZD4	8
Huiqin Yang [25]	2012	China	49	11	LRP5, FZD4	8
Ming-hui Qin [26]	2005	Japan	56	14	LRP5, FZD4	9
Jeyabalan Nallathambi [27]	2006	India	53	3	FZD4	9
Carmel Toomes [28]	2004	America	40	8	FZD4	9
Giancarlo Iarossi [29]	2017	Italy	8	6	LRP5, FZD4, NDP, TSPAN12	9
Xiao-Yan Huang [30]	2017	China	10	5	LRP5, FZD4, TSPAN12	9
Sarah Hull [31]	2019	England	10	6	LRP5, FZD4, NDP, TSPAN12, ZNF408, KIF11	8
Qi Rui [32]	2019	China	5	2	LRP5, FZD4, NDP, TSPAN12	9
Miao Tang [4]	2016	China	100	21	FZD4	9
Miao Tang [33]	2017	China	100	23	LRP5, NDP, TSPAN12	9
Kimberly A. Drenser [34]	2009	America	63	9	FZD4	9
William Carrera [35]	2021	America	-	7	LRP5, FZD4	8
Li-Yun Jia [36]	2010	China	48	15	FZD4	9
Li-Yun Jia [37]	2021	China	33	4	NDP	9
Yu Xu [38]	2014	China	85	3	TSPAN12	9

While there was extensive heterogeneity in sample characteristics and experimental conditions in the included studies. It comes from the difference of the studied population, detection methods, and target genes in each study. In addition, the phenotypes of most inherited diseases are caused by the interaction of genetic and environmental factors. Moreover, the problem of sample dropping exists in the statistical process of the included studies.

### Statistics on the frequency of gene mutations, gender and age distribution

To obtain statistically significant results on the frequency of mutations, we collected 3069 FEVR patients, and the genes of LRP5, FZD4, NDP, TSPAN12, ZNF408, KIF11 were included.

According to our statistics, LRP5 and FZD4 genes are the capital virulence genes of FEVR accounting for 13.6% (95% CI 10.9–16.3%) (S1A Fig) and 11.5% (95% CI 10.3–12.8%) (S1B Fig), respectively. The ratio of cases with TSPAN12, KIF11, NDP, and ZNF408 mutations was relatively small, 6.7% (95% CI 5.7–7.6) (S1D Fig), 5.7% (95% CI 2.8–8.7%) (S1F Fig), 4.6% (95% CI 2.8–6.4%) (S1C Fig), and 1.6% (95% CI 1.0–2.2%) (S1E Fig), respectively. All of the above patients were recorded with single gene mutation (Table 2).

**Table 2:** Frequency of virulence genes of FEVR in the large sample size.

Gene	No. of studies	Proportions	Heterogeneity	
		Risk gene frequency(95%CI)	P. Value	I^2^ Value
LRP5	15	13.6 (10.9–16.3)	0.01	54%
FZD4	20	11.5 (10.3–12.8)	< 0.01	48%
NDP	15	4.6 (2.8–6.4)	< 0.01	67%
TSPAN12	16	6.7 (5.7–7.6)	0.73	0%
ZNF408	10	1.6 (1.0–2.2)	0.6	0%
KIF11	5	5.7 (2.7–8.7)	< 0.01	81%

**Fig 2:**
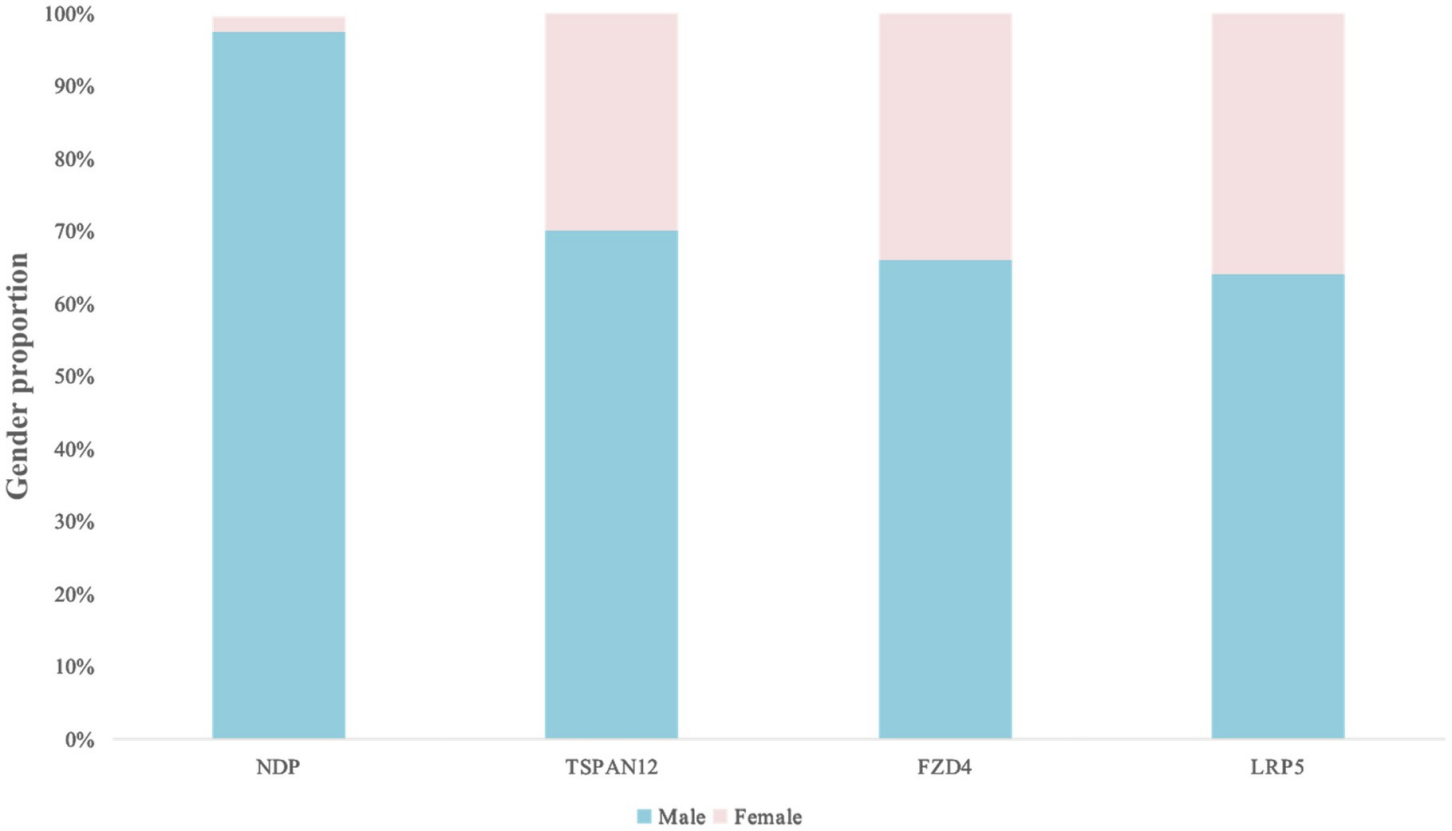
Different gender distribution in varied gene mutations.

16 studies provided the information of gender for patients with LRP5, FZD4, NDP, TSPAN12 mutation (Fig 2). The proportion of male patients is much higher than female patients. Meanwhile, almost all the probands with NDP mutations were male, which was significantly different from the distribution of patients with the other three gene mutations. Among patients with LRP5, FZD4, and TSPAN gene mutations, there is no significant difference in gender distribution. (p = 0.5 and p = 0.8).

**Fig 3:**
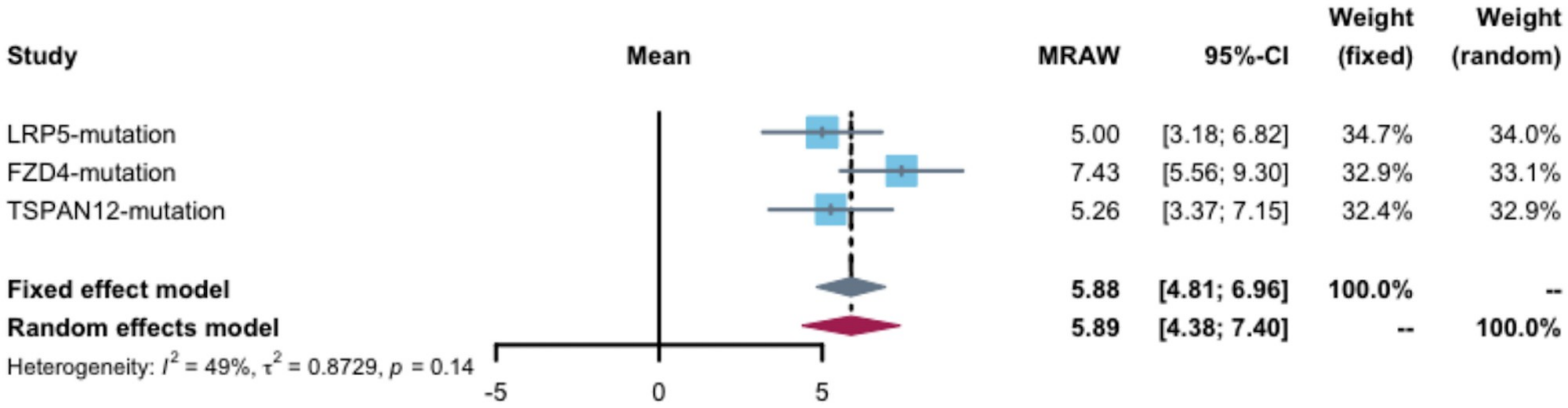
The forest plots of the age statistics for FEVR with LRP5, FZD4 and TSPAN12 gene mutations.

We also performed the statistical analysis of onset age for gene mutations carriers. The average onset age in patients with NDP mutations is 3.0 (95% CI 1.3–4.9) years which is significantly small than the patients with patients of other gene mutations. In contrast, the average onset age of cases with LRP5, FZD4, and TSPAN12 gene mutations is 5.0 (95% CI 3.2–6.8), 7.4 (95% CI 5.6–9.3), and 5.3 (95% CI 3.4–7.2), respectively that were no significant difference (Fig 3).

### Correlation between clinical staging and manifestations with gene mutation types

17 studies reported clinical stages of 269 patients with FEVR, and 18 studies focused on the ocular abnormal of 319 patients. Analysis of gene mutation types and FEVR staging is shown in Table 3 and S3 Table. We also studied the correlation between gene mutation types and clinical manifestations as shown in S4 Table. Detailed information about clinical manifestations and gene mutations of each patient is described in S5 Table.

**Table 3:** Meta-analysis of patients with mild and severe FEVR.

Gene	Mild			Sever		
	95%CI	P. Value	I^2^ Value	95%CI	P. Value	I^2^ Value
LRP5	28.1(8.7–47.5)	< 0.01	75%	71.9(52.5–91.3)	< 0.01	75%
FZD4	21.4(9.8–33.1)	0.03	52%	78.6(66.9–90.2)	0.03	52%
NDP	12.1(0.0–27.1)	0.06	52%	88.8(79.2–98.4)	0.18	34%
TSPAN12	30.6(18.3–42.9)	0.43	0%	69.4(57.1–81.7)	0.10	43%

#### LRP5

**Fig 4:**
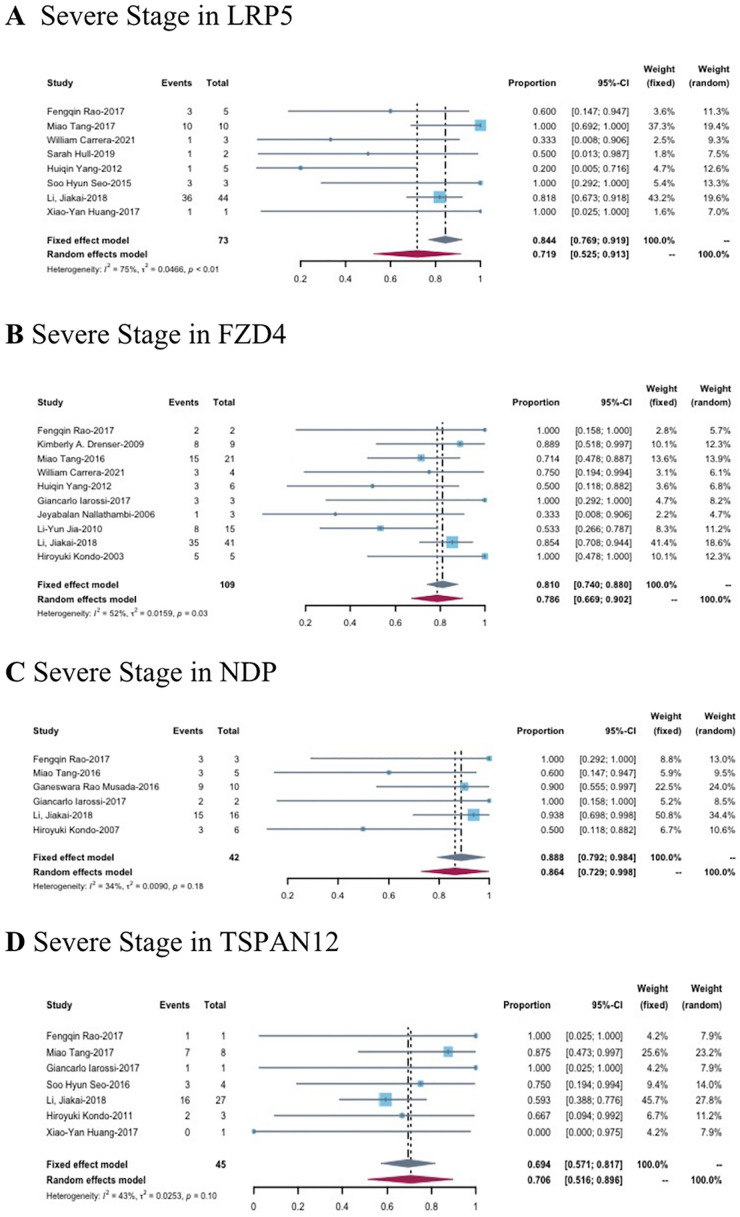
The forest plots of the proportion of patients with severe clinical manifestations in each gene mutation types.

Ten studies reported 73 patients with the LRP5 mutation, with 28.1% (95% CI 8.7–47.5%, p< 0.01) patients in mild FEVR (stage 1–2), and 71.9% (95% CI 52.5–91.3%, p< 0.01) patients in severe FEVR (stage 3–5) (Fig 4A). There are seven studies described detailed clinical manifestations of 174 patients with LRP5.

Retinal detachment was found to be the most common clinical symptom among all patients, accounting for 51.9% (95% CI 25.5–78.4%, P < 0.01) (S2A Fig). In addition, 29.5% (95% CI 14.1–45.0%, P < 0.01) (S2B Fig) patients present with retinal folds. Avascular areas were found in 28.1% (95% CI 10.6–45.5%, P < 0.01) (S2C Fig) patients. 20.9% (95% CI 1.6–40.2%, P < 0.01) (S2D Fig) patients had retrolental fibroplasia and there are 15.6% (95% CI 10.3–21.0%, P = 0.28) (S2E Fig) patients had ocular manifestations of complete retinal detachment.

#### FZD4

A total of 109 patients with clinical staging information developed FZD4 gene mutation. Among them, 21.4% (95% CI 9.8–33.1%, p = 0.03) patients are in mild FEVR (stage1-2) and 78.6% (95% CI 66.9–90.2%, p = 0.03) (Fig 4B) are the severe patients (stage 3–5). Eleven studies reported the detailed clinical manifestations of 230 patients with FZD4 gene mutation. In these cases, the avascular area is the most common symptom, accounting for 41.2% (95% CI 22.0–60.3%, p<0.01) (S3A Fig) of all enrolled patients. 30.1% (95% CI 24.4–35.8%, p = 0.06) (S3B Fig) patients had retinal folds; 15.6% (95% CI 6.1–25.1%, p<0.01) (S3C Fig) patients present with macular ectopic; Neovascularization occurred in 11.1% (95% CI 2.9–19.2%, p<0.01) (S3D Fig) of patients and there are 8.8% (95% CI 0.9–7.0%, p = 0.04) (S3E Fig) patients had complete retinal detachment.

#### NDP

Six studies described the clinical staging of 42 patients with gene mutation of NDP, one of which has missing information. Most patients are in severe FEVR (stage 3–5) accounting for 88.8% (95% CI 79.2–98.4%, p = 0.18) (Fig 4C), and only 12.1% (95% CI 0.0–27.1%, p = 0.06) cases in mild FEVR (stage 1–2). As a result, 55.7% (95% CI 27.8–83.7%, p< 0.01) (S4A Fig) patients have retinal detachment. Avascular areas were present in 19.3% (95% CI 5.7–32.9%, P < 0.01) (S4B Fig) patients. Macular displacement occurred in 18.1% (95% CI 3.7–32.5%, P < 0.01) (S4C Fig). 18.0% (95% CI 1.3–34.8%, P < 0.01) (S4D Fig) patients had retrolental fibroplasia; Retinal folds occurred in 5.5% (95% CI 0.0–12.9%, P = 0.04) patients (S4E Fig).

#### TSPAN12

Seven studies reported the clinical staging of 7 patients with gene mutation of TSPAN12. There are 30.6% (95% CI 18.3–42.9%, p = 0.43) patients in mild FEVR (stage 1–2) and 69.4% (95% CI 57.1–81.7%, p = 0.10) (Fig 4D) patients in severe FEVR (stage 3–5). The clinical manifestations of 132 patients with TSPAN12 were obtained from 8 studies. Retinal fold is the most common clinical manifestation in this case, accounting for 57.3% (95% CI 30.7–83.8%, p<0.01) (S5A Fig) of all included patients. In addition, 35.7% (95% CI 8.4–63.1%, p<0.01) (S5B Fig) patients had avascular areas. Patients with complete retinal detachment accounted for 11.8% (95% CI 6.1–17.4%, p = 0.19) (S5C Fig). 11.7% (95% CI 1.6–21.5%, p = 0.02) (S5D Fig) patients developed macular ectopic and 8.8% (95% CI 0.0–19.8%, p<0.01) (S5E Fig) patients present with neovascularization.

## Discussion

FEVR is a serious hereditary retinal disease characterized by abnormal retinal vascular development [39, 40]. Early diagnosis of FEVR is of great clinical significance but difficult, as most patients are often found after the occurrence of strabismus, nystagmus and other complications. The disease progresses rapidly in some cases and can lead to irreversible visual impairment without timely intervention. The clinical manifestations of FEVR are diverse [5] and often accompanied by atypical symptoms such as retinal hiatus and macular anterior membrane, resulting in a high misdiagnosis rate [41]. Genetic diagnosis can not only improve the accuracy of early diagnosis but also provide meaningful genetic counseling and lifelong monitoring for patients with positive family history, so as to prevent the development of the disease [42].

Cases with FEVR are relatively rare in clinical practice, so the sample size in single study is limited [40, 43], resulting in the contingency of results. To draw convincing and reliable conclusions, we integrated a large number of high-quality studies, and the total sample size included is more than 3000 cases. Our statistical analysis (Table 2) results are generally consistent with the studies by Salvo, J et al. [18] and Tian Tian T et al. [13]. Salvo, J conducted a cohort study on 92 probands and found that the patients with LRP5 and FZD4 gene mutations accounted for the largest proportion (19% and 15% respectively). The cohort study of 516 probands by Tian T et al. also found that patients with genes mutations of LRP5 and FZD4 are the most common cases accounted for 20.1% and 14.5%, respectively.

Previous studies [44–46] have found that FEVR patients of the same family and genetic mutation have different clinical manifestations. These studies believed that there was no correlation between gene mutation types and clinical phenotype [25]. However, some studies [7, 39] have suggested the correlation between gene mutation types and clinical phenotype. The above contradictory conclusions are due to the insufficient sample size of each study, so statistically significant results cannot be obtained. In our study, we integrated a large number of patients with gene mutation from high-quality studies. The sample size in our study is sufficient to draw statistical conclusions about the association between gene mutation types and clinical phenotypes. According to our statistical analysis, it was found that the most common clinical manifestation of patients with LRP5 and NDP gene mutations was retinal detachment, which was the main cause of vision loss in FEVR. In this case, surgical treatment is often adopted to save the patient’s vision. However, there are great uncertainties in the efficacy and risks of surgery. For patients in the different stages of disease, varied treatment methods are adopted [40]. Scleral buckling is generally recommended for patients in stage 3, while vitrectomy is mainly used for stages 4 and 5. We found that the patients with LRP5 mutation are mostly in stage 4 (31.51%), and NDP mutation is common in patients at stage 5 (69.05%). Studying for 105 probands, Shiyuan Wang et al. [47] came to a similar conclusion with us that most patients with LRP5 gene mutation were in stage 4, accounting for 48.39%, and 83.33% of patients with NDP gene mutation had reached stage 5 for the first visit. In terms of clinical staging and gene mutation types, a consistent overall trend was found by Shiyuan Wang and our study. That is, patients with LRP5 and NDP gene mutations suffer mostly in 4 and 5 clinical staging respectively. To this end, we suggest that once the patient is found with LRP5 and NDP mutations, it is necessary to always be alert to the occurrence of severe retinal detachment and lifelong monitoring should be carried out. Our studies suggested that the severity of cases with gene mutations is varied. Among the cases with NDP gene mutation, the proportion of severe patients was the highest (88.8%). The severity of clinical phenotype for patients with FZD4 gene mutation is second only to NDP, and about 78.6% of patients showed severe symptoms at the first visit. The patients with LRP5 and TSPAN12 gene mutations show relatively mild clinical phenotypes, which is consistent with the conclusion of Li Jiakai et al. [3]. Among the 389 patients with single gene mutation, all NDP mutation carriers are in stage 5. 86.2% and 51.6% of patients with LRP5 and FZD4 gene mutations suffer the clinical manifestations of stage 4 or 5, respectively. None of the patients with TSPAN12 mutation is in stage 5. In summary, patients with NDP gene mutation suffer the most severe clinical manifestations, followed by FZD4 and LRP5 are lower. The patients with TSPAN12 gene mutation have milder clinical symptoms than the other three gene mutations carriers.

**Fig 5:**
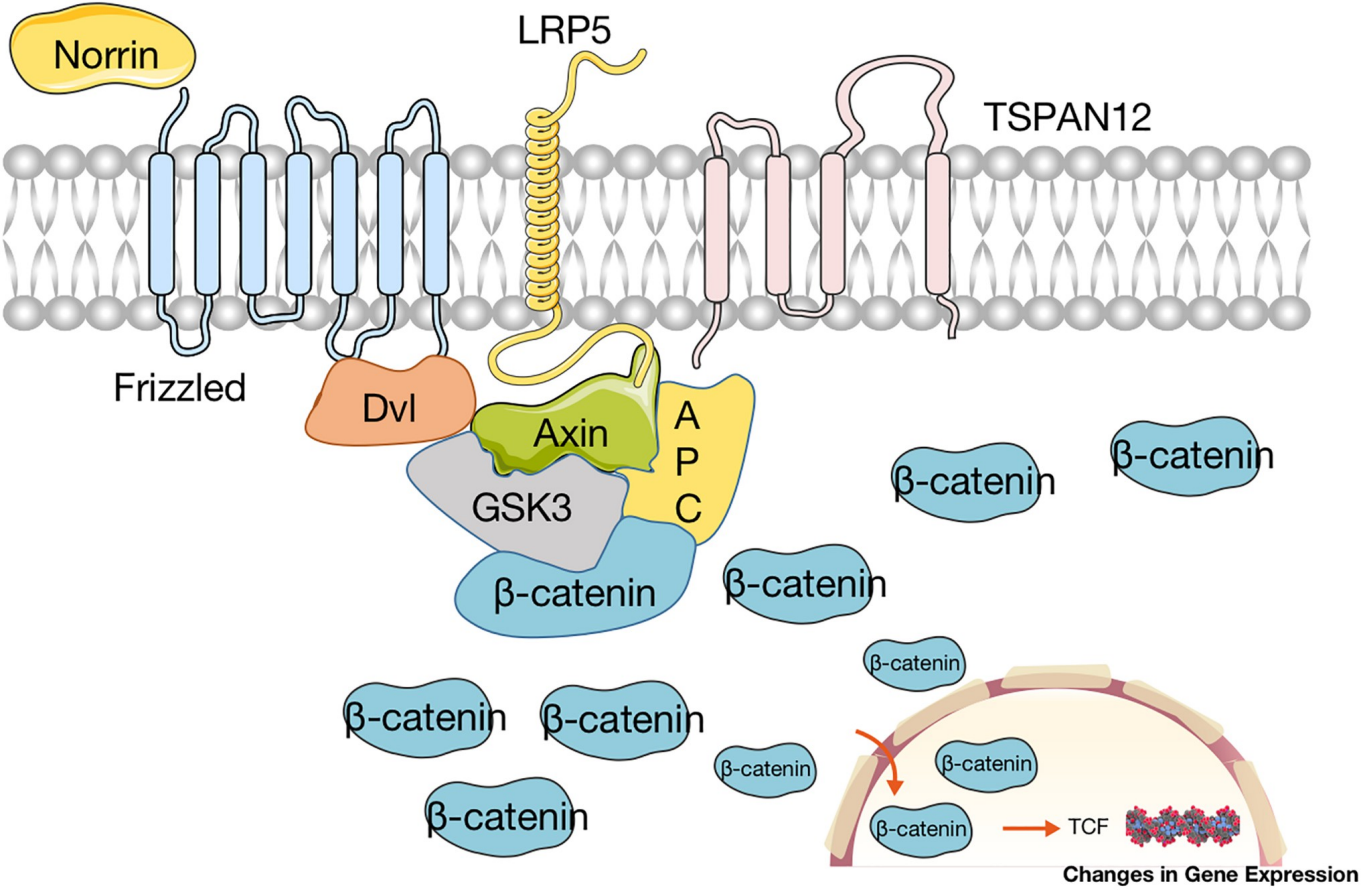
Schematic diagram of the Norrin/β-catenin signal pathway. The Norrie protein encoded by NDP first binds to FZD4 and then forms a terpolymer complex with coreceptor LRP5, TSPAN12 as an auxiliary component of the complex. Activated FZD4 bound Dvl and phosphorylated LRP5 recruited Axin, GSK-3, APC to the plasma membrane, resulting in the suppression of β-catenin phosphorylation degradation. When the amount of β-catenin accumulates to a certain extent in cytoplasmic, part of β-catenin is translocated to the nucleus and binds to the TCF to changes in gene expression.

LRP5, FZD4, NDP, and TSPAN12 are recognized as the main pathogenic genes of FEVR [48]. A large number of studies [49] have been devoted to exploring the mechanism of these four genes in the development of retinal blood vessels. The present study found that these four genes all interfere with the development of retinal vascular endothelial cells by affecting the Norrin/β-catenin signaling pathway, leading to the occurrence of FEVR [8]. The Norrin/β-catenin signaling pathway is a signaling pathway activated by binding of FZD4 with NDP as ligand [50]. In this process (Fig 5), the Norrie protein encoded by NDP first binds to FZD4, and then forms a terpolymer complex with coreceptor LRP5, TSPAN12 as an auxiliary component of the complex, activates the Norrin/β-catenin signaling together with LRP5, FZD4, and NDP. It promotes the accumulation and stable existence of β-catenin in cytoplasmic cells. When the amount of β-catenin accumulates to a certain extent, part of β-catenin is translocated to the nucleus and binds to the LEF/TCF transcription factor family to initiate transcription of downstream β-catenin signals.

Multiple genes play different functions in the signaling pathway and bring varied effects on the Norrin/β-catenin signaling pathway. As a result, the amount of β-catenin that eventually enters the nucleus is also different, which is the main factor affecting the severity of the disease [39]. As a specific ligand, the binding of NDP to the FZD4 receptor is the primary condition for the initiation of the Norrin/β-catenin signaling pathway. Norrin/β-catenin signaling can be initiated only when NDP is normal. FZD4 acts as a receptor for Norrie. Its activation is also a necessary condition for the activation of the Norrin/β-catenin pathway. Only when FZD4 binds to Norrie on the membrane can Dvl which inhibits β-catenin phosphorylation be activated in the cytoplasm. LRP5 and TSPAN12 participate in the signaling pathway as co-receptors and co-components respectively, but not a decisive role. This explains why patients with NDP mutations tend to suffer the most severe clinical symptoms, followed by FZD4, while patients with LRP5 and TSPAN12 mutations have relatively mild clinical manifestations. In addition, a small number of patients with FEVR have been found two or three genetic mutations, and these cases tend to suffer more severe manifestations than patients with a single gene mutation [14, 26]. This further suggests that the clinical phenotype of FEVR may be closely related to the degree of Norrin/β-catenin signaling. Some studies have carried out animal experiments to explore the relationship between gene mutations with the occurrence and development of FEVR. It was found that in mice with NDP mutation, the development of superficial retinal blood vessels was retarded, and deep blood vessels could not form at the same time [51]. The effect of FZD4 mutation on retinal blood vessels was similar to that of NDP [52]. Compared with mice with mutations of NDP and FZD4, the mice for LRP5 gene knockout exhibited relatively mild abnormalities, with only thinning of capillaries and loss of lumen [53]. For the mice that knocked out the TSPAN12 gene, all of the above abnormalities appeared [54]. This may be due to the fact that TSPAN12 plays a catalytic role in the Norrin/β-catenin signaling pathway. The mutation of TSPAN12 reduces the effect of each gene to some extent. At present, the severity of retinal abnormalities in mice caused by TSPAN12 gene mutation is still unclear. Overall, the conclusions of our statistical analysis matched the pathogenesis explored in the current studies. Additionally, Shuai Han et al. [55] found that different gene mutations have varying degrees of effects on the activity of Norrin/β-catenin Signaling Pathway. In the future, we will further explore the correlation between gene mutations sites and clinical manifestations of cases with FEVR, and study the interaction between various mutated genes.

In addition to occasional sample drops, potential limitations of this study include the diversity of the study population, detection methods, and target genes. First, most of the subjects included in our study were Asians, so our statistical results and conclusions were limited by region and population. Second, the criteria for assessing the severity of patients with unclear disease stages are inadequate. We assessed the severity of patients based on the anatomical features and morphology of the fundus described in the included studies, while the patient’s visual function and its relation to anatomical changes were not considered. In addition, the gene types studied in this paper are not comprehensive, the genetic diversity and the interaction between multiple genes are not clear. In future research, we will conduct a more comprehensive and in-depth exploration of related gene types, pathogenic factors, and their interactions.

## Conclusion

In this paper, we firstly provide the meta-analysis of the pathogenic gene mutation frequency and the correlation between the gene mutation types with clinical manifestations from more than 3200 participants. The statistical analysis suggests that the frequency of mutations in different gene types varies in patients of FEVR, and the correlation between the gene mutation types and clinical manifestations is found. These results can assist doctors in adopting more targeted treatment and monitoring methods for patients with different gene mutations.

## Supporting information

S1 Fig Forest plot of LRP5, FZD4, NDP, TSPAN12, ZNF408 and KIF11 mutation frequencies in FEVR patients. (TIF)

S2 Fig Proportion of clinical manifestations in patients with LRP5 gene mutation. (TIF)

S3 Fig Proportion of clinical manifestations in patients with FZD4 gene mutation. (TIF)

S4 Fig Proportion of clinical manifestations in patients with NDP gene mutation. (TIF)

S5 Fig Proportion of clinical manifestations in patients with TSPAN12 gene mutation. (TIF)

S1 Table Detail search strategy of seven databases. (DOCX)

S2 Table Quality assessment of cohort studies included in the meta-analysis. The scale contains 11 items: 1-Define the source of information (survey, record review); 2- List inclusion and exclusion criteria for exposed and unexposed subjects (cases and controls) or refer to previous publications; 3- Indicate time period used for identifying patients; 4- Indicate whether or not subjects were consecutive if not population-based; 5- Indicate if evaluators of subjective components of study were masked to other aspects of the status of the participants; 6- Describe any assessments undertaken for quality assurance purposes (e.g., test/retest of primary outcome measurements); 7- Explain any patient exclusions from analysis; 8- Describe how confounding was assessed and/or controlled; 9- If applicable, explain how missing data were handled in the analysis; 10- Summarize patient response rates and completeness of data collection; 11- Clarify what follow-up, if any, was expected and the percentage of patients for which incomplete data or follow-up was obtained. The "yes", "no" and "unclear" categories are used respectively, with "1", "0" and "0" points respectively.
(DOCX)

S3 Table Probands of degree at different stages of FEVR in different gene groups. (DOCX)

S4 Table Feature of FEVR patients in different gene groups. *When the number of relevant references for a certain gene mutation is less than 3, the statistical results are considered to be no clinical significance and the gene mutation is not counted. AZ = Avascular zone; NV = Neovascularization; Exu = Exudation; ME = Macular ectopia; RLF = Retrolental fibroplasia; RF = Retinal folds; RD = Retinal detachment; TRD = Tractional retinal detachment; Fib = Fibroplasia; CRD = Complete retinal detachment.
(DOCX)

S5 Table The detailed information and clinical ophthalmological features about each proband in FEVR. *This form contains only those proband who mentioned specific information in the study. OD = Oculus Dexter; OS = Oculus Sinister; OU = Oculus Unati; M = Male; F = Female; NA = No information available; AZ = Avascular zone; NV = Neovascularization; Exu = Exudation; ME = Macular ectopia; RLF = Retrolental fibroplasia; RF = Retinal folds; RD = Retinal detachment; Fib = Fibroplasia; CRD = Complete retinal detachment; TRD = Tractional retinal detachment; ERD = Exudative retinal detachment; PHPV = Persistent Hyperplastic Primary Vitreous; VH = vitreous hemorrhage; EBV = Elongated branching of vessels; IBV = Increased branching of vessels; FRD = Falciform retinal detachment.
(DOCX)

S1 File PRISMA 2009 flow diagram. (DOC)

S2 File Meta-analysis on genetic association studies checklist. (DOCX)

S1 Checklist PRISMA 2009 checklist. (DOC)
