# Supplementary figures and images for: Genotype-phenotype associations in familial exudative vitreoretinopathy: A systematic review and meta-analysis on more than 3200 individuals
Source: PLoS One. 2022 Jul 13;17(7):e0271326. doi: 10.1371/journal.pone.0271326 (PMC9278778; doi:10.1371/journal.pone.0271326)

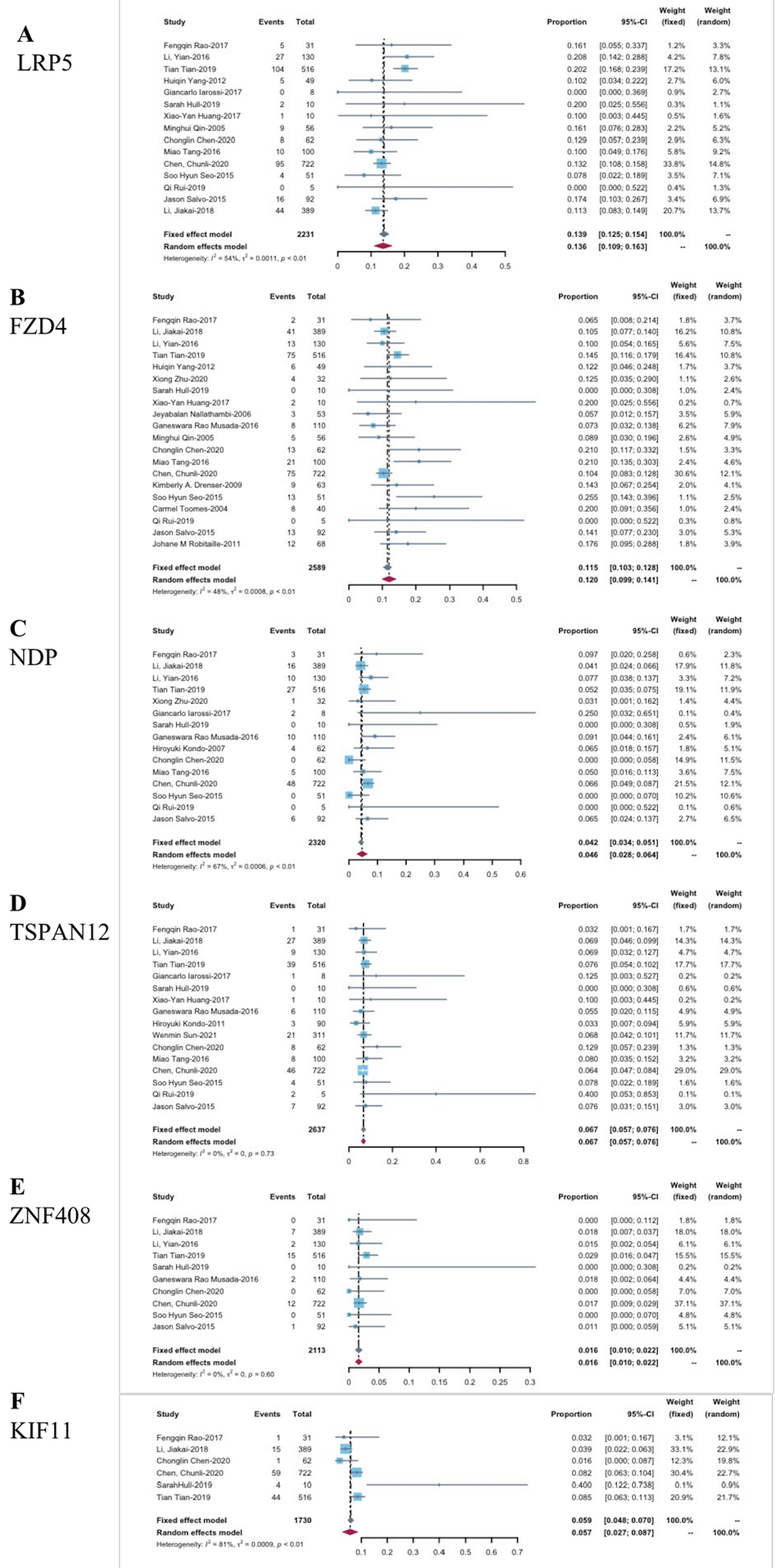

Supplement: S1 Fig — (TIF) [file pone.0271326.s001.tif]

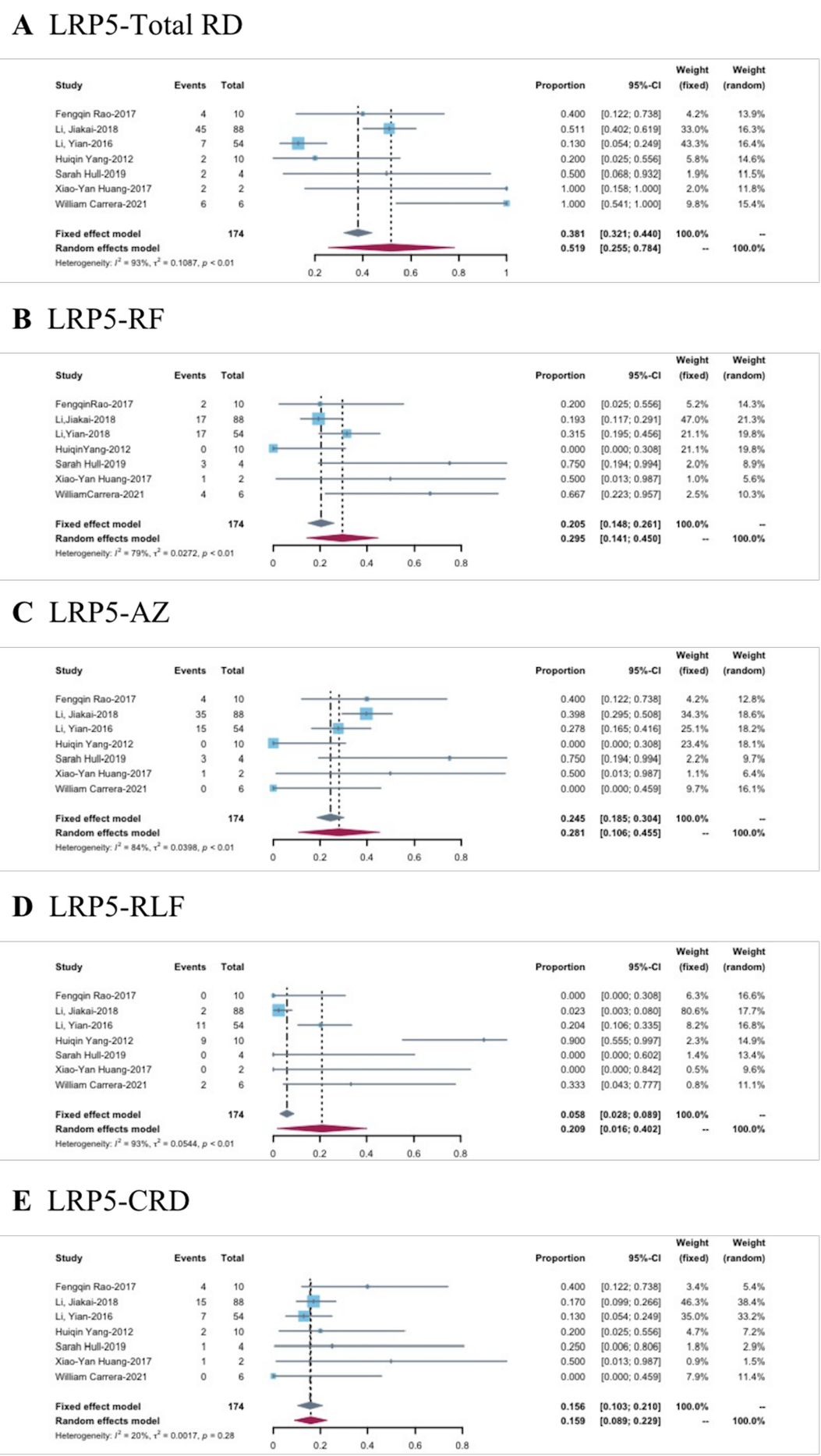

Supplement: S2 Fig — (TIF) [file pone.0271326.s002.tif]

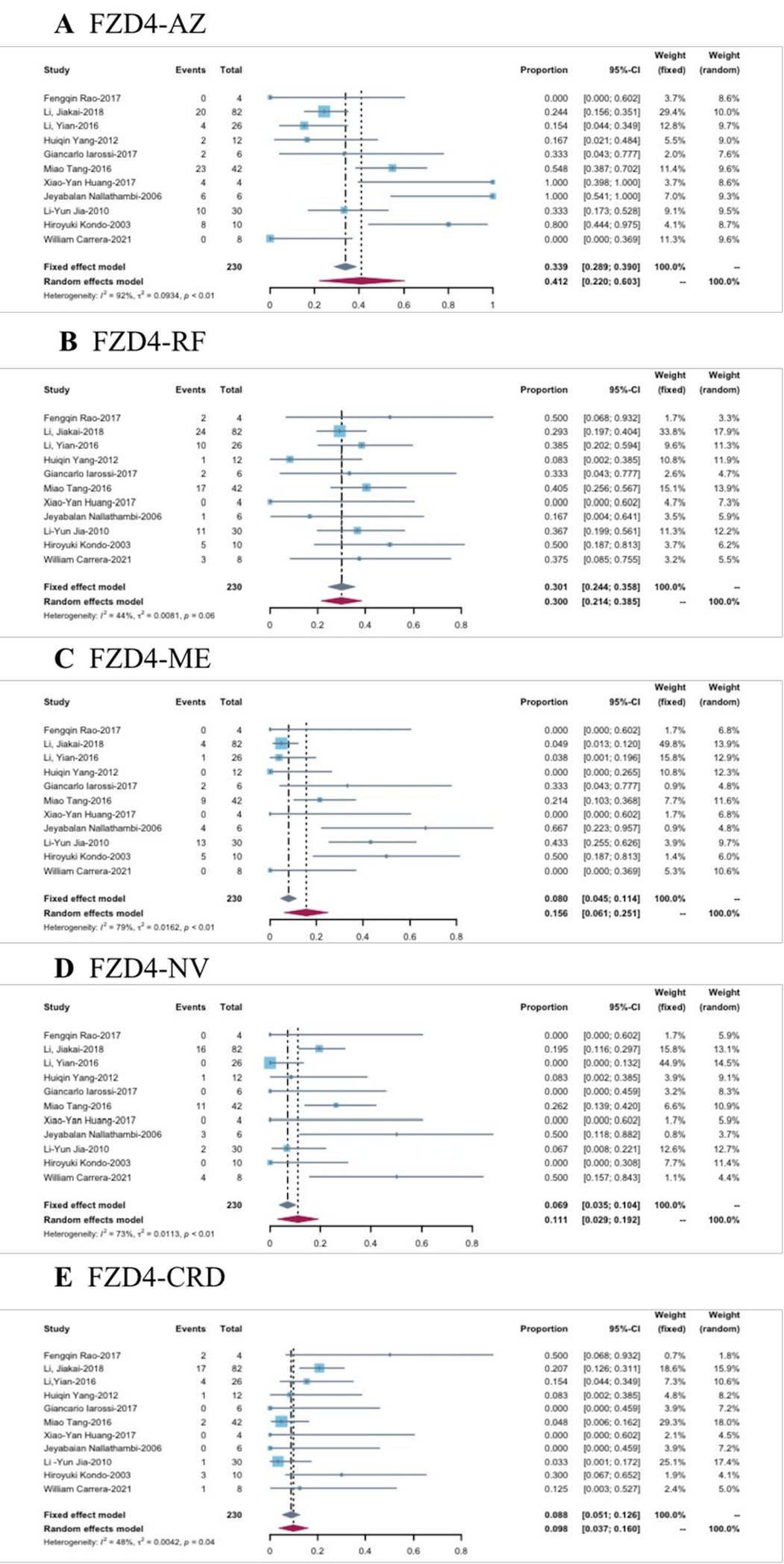

Supplement: S3 Fig — (TIF) [file pone.0271326.s003.tif]

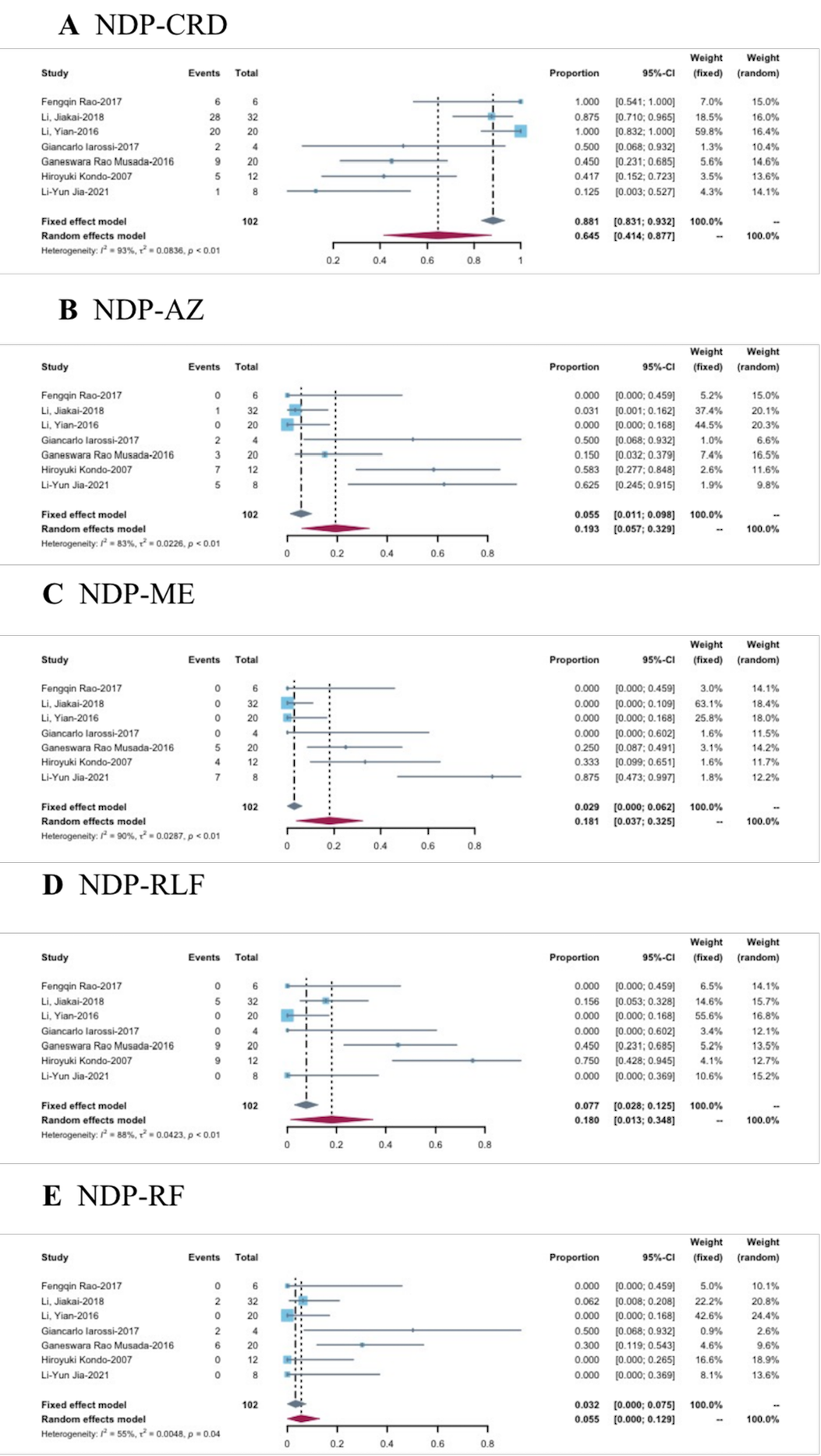

Supplement: S4 Fig — (TIF) [file pone.0271326.s004.tif]

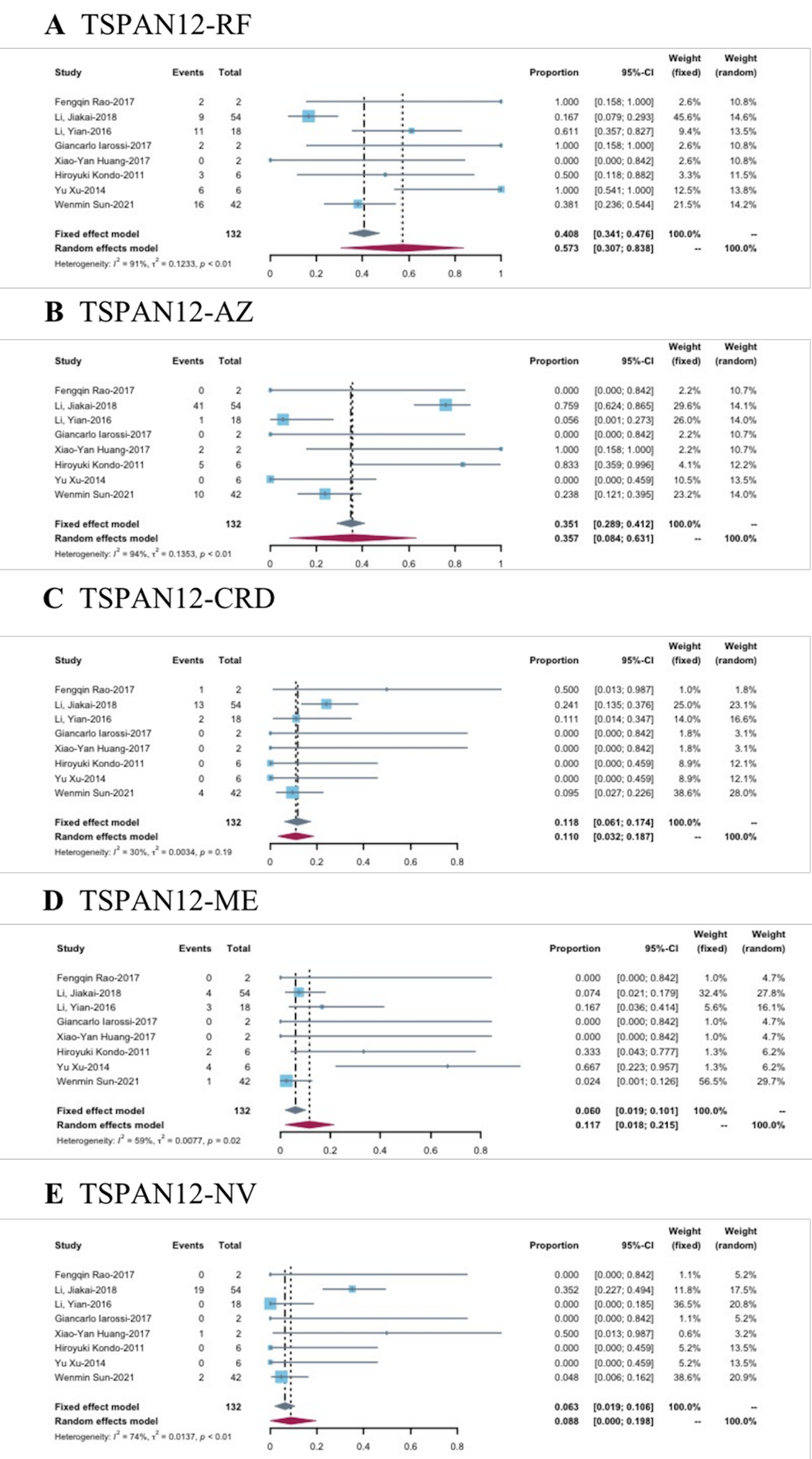

Supplement: S5 Fig — (TIF) [file pone.0271326.s005.tif]
