# Supplementary material for: Genotype-phenotype associations in familial exudative vitreoretinopathy: A systematic review and meta-analysis on more than 3200 individuals
Source: PLoS One. 2022 Jul 13;17(7):e0271326. doi: 10.1371/journal.pone.0271326 (PMC9278778; doi:10.1371/journal.pone.0271326)
Supplement: S1 Table — (DOCX) [file pone.0271326.s006.docx]

**S1 Table. Detail search strategy of seven databases**

| **Databases** | **No. of studies** | **Search strategy** |
| --- | --- | --- |
| PubMed | 188 | (("Familial Exudative Vitreoretinopathies"[1]) OR (((((((Exudative Vitreoretinopathy, Familial[Title/Abstract]) OR (Vitreoretinopathy, Familial Exudative[Title/Abstract])) OR (Familial Exudative Vitreoretinopathy[Title/Abstract])) OR (X-Linked Familial Exudative Vitreoretinopathy[Title/Abstract])) OR (X Linked Familial Exudative Vitreoretinopathy[Title/Abstract])) OR (XL-FEVR[Title/Abstract])) OR (FEVR[Title/Abstract]))) AND (("Genes"[Mesh]) OR (((((((Gene[Title/Abstract]) OR (Cistron[Title/Abstract])) OR (Cistrons[Title/Abstract])) OR (Genetic Materials[Title/Abstract])) OR (Genetic Material[Title/Abstract])) OR (Material, Genetic[Title/Abstract])) OR (Materials, Genetic[Title/Abstract]))) |
| Embase | 131 | ('familial exudative vitreoretinopathy'/exp OR 'exudative vitreoretinopathy, familial':ab,ti OR 'vitreoretinopathy, familial exudative':ab,ti OR 'familial exudative vitreoretinopathies':ab,ti OR 'x-linked familial exudative vitreoretinopathy':ab,ti OR 'x linked familial exudative vitreoretinopathy':ab,ti OR 'xl-fevr':ab,ti) AND ('gene'/exp OR 'gene':ab,ti OR 'cistron':ab,ti OR 'cistrons':ab,ti OR 'genetic materials':ab,ti OR 'genetic material':ab,ti OR 'material, genetic':ab,ti OR 'materials, genetic':ab,ti) |
| Web of Science | 480 | [(TS=(Familial Exudative Vitreoretinopathies OR Exudative Vitreoretinopathy, Familial OR Vitreoretinopathy, Familial Exudative OR Familial Exudative Vitreoretinopathy OR X-Linked Familial Exudative Vitreoretinopathy OR X Linked Familial Exudative Vitreoretinopathy OR XL-fever[Title/Abstract OR fever)) AND TS=(Genes OR Gene OR Cistron OR Cistrons OR Genetic Materials OR Genetic Material OR Material, Genetic OR Materials, Genetic)](file:////https/77726476706e69737468656265737421e7e056d230356a5f781b8aa59d5b20301c1db852/wos/alldb/summary/f2d2e5c5-861d-47d2-8f8f-d1702566e837-043bc68f/relevance/1) |
| CBM | 38 | ("家族性渗出性视网膜玻璃体病变"[常用字段] OR "家族性渗出性玻璃体视网膜病变"[常用字段]) AND ("基因"[全部字段] OR "顺反子"[全部字段] OR "基因"[主题词]) |
| CNKI | 41 | [(主题=家族性渗出性玻璃体视网膜病变) AND (主题=基因) AND (主题=临床特征) OR (主题=临床表现) OR (主题=临床表型)](https://kns.cnki.net/KNS8/AdvSearch?id=67&dbcode=SCDB&searchtype=gradeSearch&ishistory=1) |
| Wan Fang | 63 | 主题:(家族性渗出性玻璃体视网膜病变or家族性渗出性视网膜玻璃体病变) and 主题:(基因) and 主题:(临床表现or临床特征or临床表型) |
| VIP | 29 | [((题名或关键词=家族性渗出性玻璃体视网膜病变 OR 题名或关键词=家族性渗出性视网膜玻璃体病变) AND 任意字段=基因)](file:////http/77726476706e69737468656265737421e1fe4a9d297e6b41680199e29b5a2e/Qikan/search/index%3fLngMySearHistoryIdGuid=b0d1aa07-13d2-448f-a04a-deda35fbc32a&from=Qikan_Article_History) |
| Cochrane | 0 | 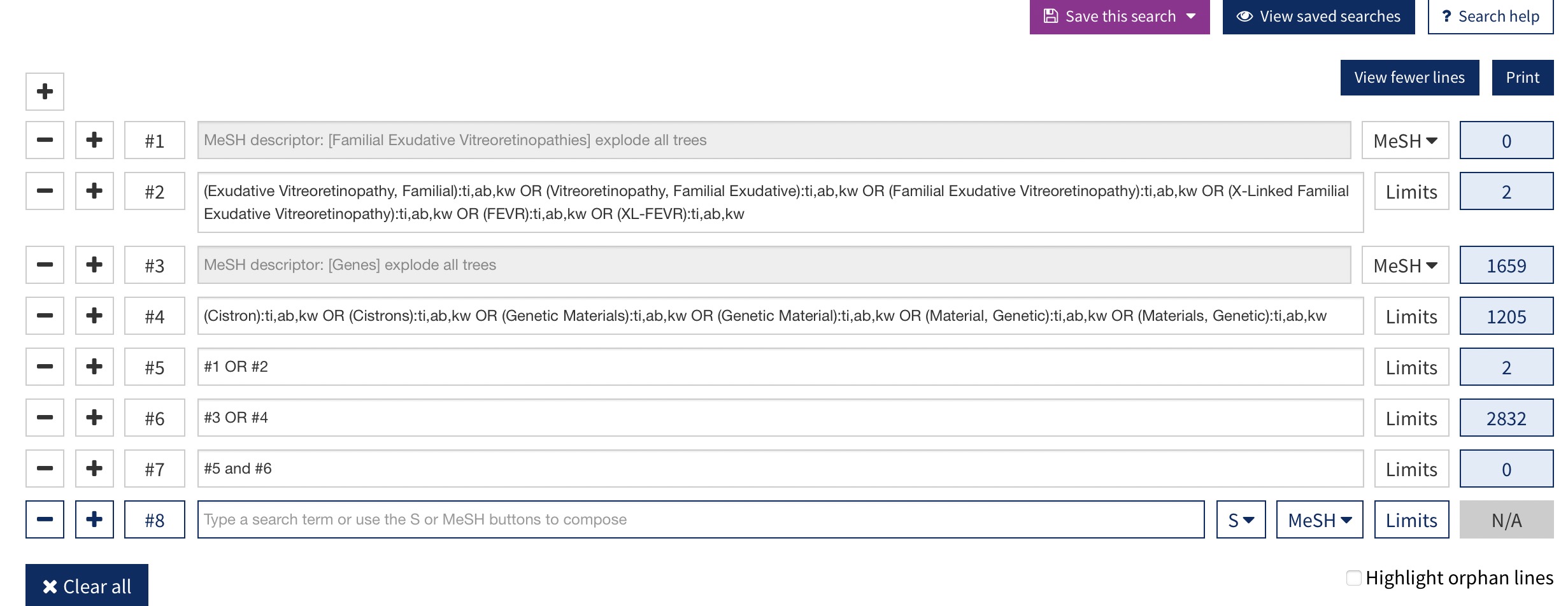 |
