# Supplementary material for: Genotype-phenotype associations in familial exudative vitreoretinopathy: A systematic review and meta-analysis on more than 3200 individuals
Source: PLoS One. 2022 Jul 13;17(7):e0271326. doi: 10.1371/journal.pone.0271326 (PMC9278778; doi:10.1371/journal.pone.0271326)
Supplement: S2 Table — The scale contains 11 items: 1-Define the source of information (survey, record review); 2- List inclusion and exclusion criteria for exposed and unexposed subjects (cases and controls) or refer to previous publications; 3- Indicate time period used for identifying patients; 4- Indicate whether or not subjects were consecutive if not population-based; 5- Indicate if evaluators of subjective components of study were masked to other aspects of the status of the participants; 6- Describe any assessments undertaken for quality assurance purposes (e.g., test/retest of primary outcome measurements); 7- Explain any patient exclusions from analysis; 8- Describe how confounding was assessed and/or controlled; 9- If applicable, explain how missing data were handled in the analysis; 10- Summarize patient response rates and completeness of data collection; 11- Clarify what follow-up, if any, was expected and the percentage of patients for which incomplete data or follow-up was obtained. The "yes", "no" and "unclear" categories are used respectively, with "1", "0" and "0" points respectively. (DOCX) [file pone.0271326.s007.docx]

**S2 Table. Quality Assessment of Cohort Studies Included in the Meta-analysis**

| **Study** | **1** | **2** | **3** | **4** | **5** | **6** | **7** | **8** | **9** | **10** | **11** |
| --- | --- | --- | --- | --- | --- | --- | --- | --- | --- | --- | --- |
| Feng-Qin Rao et al,2017 [1] | 1 | 1 | 0 | 1 | 1 | 1 | 1 | 1 | 1 | 1 | 0 |
| Chen, Chunli et al,2020 [2] | 1 | 1 | 1 | 1 | 1 | 1 | 0 | 1 | 1 | 1 | 0 |
| Tian, T et al,2019 [3] | 1 | 1 | 1 | 1 | 1 | 1 | 1 | 1 | 1 | 1 | 0 |
| Li, J.-K et al,2018 [4] | 1 | 1 | 1 | 1 | 1 | 1 | 0 | 1 | 1 | 1 | 0 |
| Li, Yian et al,2016 [5] | 1 | 1 | 1 | 1 | 1 | 1 | 1 | 1 | 1 | 1 | 0 |
| Chen，C et al,2020 [6] | 1 | 1 | 0 | 0 | 1 | 1 | 1 | 1 | 1 | 1 | 1 |
| Sun, W et al,2021 [7] | 1 | 1 | 0 | 1 | 1 | 1 | 1 | 1 | 0 | 1 | 0 |
| Musada,G.R et al,2016 [8] | 1 | 1 | 0 | 1 | 1 | 1 | 1 | 1 | 1 | 1 | 0 |
| Musada,G.R et al,2016 [9] | 1 | 1 | 0 | 1 | 1 | 1 | 1 | 1 | 1 | 1 | 0 |
| Zhu, X et al,2020 [10] | 1 | 1 | 0 | 1 | 1 | 1 | 1 | 1 | 1 | 1 | 0 |
| Salvo, J et al,2015 [11] | 1 | 1 | 1 | 1 | 1 | 1 | 1 | 1 | 1 | 1 | 0 |
| Seo, S.H et al,2015 [12] | 1 | 1 | 1 | 1 | 1 | 1 | 1 | 1 | 1 | 1 | 1 |
| Seo, S.H et al,2016 [13] | 1 | 1 | 1 | 1 | 1 | 1 | 1 | 1 | 1 | 1 | 1 |
| Kondo, H et al,2003 [14] | 1 | 1 | 0 | 1 | 1 | 1 | 1 | 1 | 1 | 1 | 0 |
| Kondo, H et al,2011 [15] | 1 | 1 | 0 | 1 | 1 | 1 | 1 | 1 | 1 | 1 | 0 |
| Kondo, H., et al,2007 [16] | 1 | 1 | 0 | 1 | 1 | 1 | 1 | 1 | 1 | 1 | 0 |
| Robitaille, J.M et al,2011 [17] | 1 | 1 | 0 | 1 | 1 | 1 | 1 | 1 | 1 | 1 | 0 |
| Yang, H et al,2012 [18] | 1 | 1 | 0 | 1 | 1 | 1 | 1 | 0 | 1 | 1 | 0 |
| Qin, M.H et al,2005 [19] | 1 | 1 | 0 | 1 | 1 | 1 | 1 | 1 | 1 | 1 | 0 |
| Nallathambi, J et al,2006 [20] | 1 | 1 | 0 | 1 | 1 | 1 | 1 | 1 | 1 | 1 | 0 |
| Toomes, C et al,2004 [21] | 1 | 1 | 0 | 1 | 1 | 1 | 1 | 1 | 1 | 1 | 0 |
| Iarossi, G et al,2017 [22] | 1 | 1 | 0 | 1 | 1 | 1 | 1 | 1 | 1 | 1 | 0 |
| Huang, X.-Y et al,2017 [23] | 1 | 1 | 0 | 1 | 1 | 1 | 1 | 1 | 1 | 1 | 0 |
| Hull, S et al,2019 [24] | 1 | 1 | 0 | 0 | 1 | 1 | 1 | 1 | 1 | 1 | 0 |
| Qi Rui et al,2019 [25] | 1 | 1 | 1 | 1 | 1 | 1 | 1 | 1 | 1 | 0 | 0 |
| Tang, M et al,2016[26] | 1 | 1 | 0 | 1 | 1 | 1 | 1 | 1 | 1 | 1 | 0 |
| Tang, M et al,2017 [27] | 1 | 1 | 0 | 1 | 1 | 1 | 1 | 1 | 1 | 1 | 0 |
| Drenser, K.A et al,2009 [28] | 1 | 1 | 0 | 1 | 1 | 1 | 1 | 1 | 1 | 1 | 0 |
| Carrera, W et al,2021 [29] | 1 | 1 | 0 | 1 | 1 | 1 | 1 | 1 | 0 | 1 | 0 |
| Jia, L.-Y et al,2010 [30] | 1 | 1 | 0 | 1 | 1 | 1 | 1 | 1 | 1 | 1 | 0 |
| Jia, L.-Y. and K. Ma,2021 [31] | 1 | 1 | 0 | 1 | 1 | 1 | 1 | 1 | 1 | 1 | 0 |
| Xu, Y et al,2014 [32] | 1 | 1 | 0 | 1 | 1 | 1 | 1 | 1 | 1 | 1 | 0 |

The scale contains 11 items: 1-Define the source of information (survey, record review); 2- List inclusion and exclusion criteria for exposed and unexposed subjects (cases and controls) or refer to previous publications; 3- Indicate time period used for identifying patients; 4- Indicate whether or not subjects were consecutive if not population-based; 5- Indicate if evaluators of subjective components of study were masked to other aspects of the status of the participants; 6- Describe any assessments undertaken for quality assurance purposes (e.g., test/retest of primary outcome measurements); 7- Explain any patient exclusions from analysis; 8- Describe how confounding was assessed and/or controlled; 9- If applicable, explain how missing data were handled in the analysis; 10- Summarize patient response rates and completeness of data collection; 11- Clarify what follow-up, if any, was expected and the percentage of patients for which incomplete data or follow-up was obtained.The "yes", "no" and "unclear" categories are used respectively, with "1", "0" and "0" points respectively.

**Reference**

1. Rao F-Q, Cai X-B, Cheng F-F, Cheng W, Fang X-L, Li N, et al. Mutations in LRP5, FZD4, TSPAN12, NDP, ZNF408, or KIF11 Genes Account for 38.7% of Chinese Patients With Familial Exudative Vitreoretinopathy. Invest Ophthalmol Vis Sci. 2017 May 1;58(5):2623-2629. PubMed PMID: 28494495.

2. Chunli Chen. Clinical Characteristics and Genetic Diversity of Familial Exudative Vitreoretinopathy [dissertation], Tianjin Medical University; 2020.

3. Tian T, Chen C, Zhang X, Zhang Q, Zhao P. Clinical and Genetic Features of Familial Exudative Vitreoretinopathy With Only-Unilateral Abnormalities in a Chinese Cohort. JAMA Ophthalmol. 2019 Sep 1;137(9):1054-1058. PubMed PMID: 31169861; PubMed Central PMCID: PMC6555475.

4. Li J-K, Li Y, Zhang X, Chen C-L, Rao Y-Q, Fei P, et al. Spectrum of Variants in 389 Chinese Probands With Familial Exudative Vitreoretinopathy. Invest Ophthalmol Vis Sci. 2018 Nov 1;59(13):5368-5381. PubMed PMID: 30452590.

5. Yian Li, Qi Zhang, Jing Li Qiujing Huang, Peiquan Zhao. Phenotype-Genotype in Familial Exudative Vitreoretinopathy [dissertation], Shanghai Jiao Tong University; 2016.

6. Chen C, Sun L, Li S, Huang L, Zhang T, Wang Z, et al. The spectrum of genetic mutations in patients with asymptomatic mild familial exudative vitreoretinopathy. Exp Eye Res. 2020 Mar;192:107941. PubMed PMID: 31987760.

7. Sun W, Xiao X, Li S, Jia X, Wang P, Zhang Q. Pathogenic variants and associated phenotypic spectrum of TSPAN12 based on data from a large cohort. Graefes Arch Clin Exp Ophthalmol. 2021 Oct;259(10):2929-2939. PubMed PMID: 33907885.

8. Musada GR, Syed H, Jalali S, Chakrabarti S, Kaur I. Mutation spectrum of the FZD-4, TSPAN12 AND ZNF408 genes in Indian FEVR patients. BMC Ophthalmol. 2016 Jun 17;16:90. PubMed PMID: 27316669; PubMed Central PMCID: PMC4912735.

9. Musada GR, Jalali S, Hussain A, Chururu AR, Gaddam PR, Chakrabarti S, et al. Mutation spectrum of the Norrie disease pseudoglioma (NDP) gene in Indian patients with FEVR. Mol Vis. 2016 May 16;22:491-502. PubMed PMID: 27217716; PubMed Central PMCID: PMC4872281.

10. Zhu X, Sun K, Huang L, Ma S, Hao F, Yang Z, et al. Identification of Novel Mutations in the FZD4 and NDP Genes in Patients with Familial Exudative Vitreoretinopathy in South India. Genet Test Mol Biomarkers. 2020 Feb;24(2):92-98. PubMed PMID: 31999491.

11. Salvo J, Lyubasyuk V, Xu M, Wang H, Wang F, Nguyen D, et al. Next-Generation Sequencing and Novel Variant Determination in a Cohort of 92 Familial Exudative Vitreoretinopathy Patients. Invest Ophthalmol Vis Sci. 2015 Feb 24;56(3):1937-46. PubMed PMID: 25711638; PuMed Central PMCID: PMC4365990.

12. Seo SH, Yu YS, Park SW, Kim JH, Kim HK, Cho SI, et al. Molecular Characterization of FZD4, LRP5, and TSPAN12 in Familial Exudative Vitreoretinopathy. Invest Ophthalmol Vis Sci. 2015 Aug;56(9):5143-51. PubMed PMID: 26244290.

13. Seo SH, Kim MJ, Park SW, Kim JH, Yu YS, Song JY, et al. Large Deletions of TSPAN12 Cause Familial Exudative Vitreoretinopathy (FEVR). Invest Ophthalmol Vis Sci. 2016 Dec 1;57(15):6902-6908. PubMed PMID: 28002565.

14. Kondo H, Hayashi H, Oshima K, Tahira T, Hayashi K. Frizzled 4 gene (FZD4) mutations in patients with familial exudative vitreoretinopathy with variable expressivity. Br J Ophthalmol. 2003 Oct;87(10):1291-5. PubMed PMID: 14507768; PubMed Central PMCID: PMC1920788.

15. Kondo H, Kusaka S, Yoshinaga A, Uchio E, Tawara A, Hayashi K, et al. Mutations in the TSPAN12 gene in Japanese patients with familial exudative vitreoretinopathy. Am J Ophthalmol. 2011 Jun;151(6):1095-1100.e1. PubMed PMID: 21334594.

16. Kondo H, Qin M, Kusaka S, Tahira T, Hasebe H, Hayashi H, et al. Novel mutations in Norrie disease gene in Japanese patients with Norrie disease and familial exudative vitreoretinopathy. Invest Ophthalmol Vis Sci. 2007 Mar;48(3):1276-82. PubMed PMID: 17325173.

17. Robitaille JM, Zheng B, Wallace K, Beis MJ, Tatlidil C, Yang J, et al. The role of Frizzled-4 mutations in familial exudative vitreoretinopathy and Coats disease. Br J Ophthalmol. 2011 Apr;95(4):574-9. PubMed PMID: 21097938.

18. Yang H, Li S, Xiao X, Wang P, Guo X, Zhang Q. Identification of FZD4 and LRP5 mutations in 11 of 49 families with familial exudative vitreoretinopathy. Mol Vis. 2012;18:2438-46. Epub 2012 Oct 4. PubMed PMID: 23077402; PuMed Central PMCID: PMC3472927.

19. Qin MH, Hayashi H, Oshima K, Tahira T, Hayashi K, Kondo H. Complexity of the genotype-phenotype correlation in familial exudative vitreoretinopathy with mutations in the LRP5 and/or FZD4 genes. Hum Mutat. 2005 Aug;26(2):104-12. PubMed PMID: 15981244.

20. Nallathambi J, Shukla D, Rajendran A, Namperumalsamy P, Muthulakshmi R, Sundaresan P. Identification of novel FZD4 mutations in Indian patients with familial exudative vitreoretinopathy. Mol Vis. 2006 Sep 21;12:1086-92. PubMed PMID: 17093393.

21. Toomes C, Bottomley HM, Scott S, Mackey DA, Craig JE, Appukuttan B, et al. Spectrum and frequency of FZD4 mutations in familial exudative vitreoretinopathy. Invest Ophthalmol Vis Sci. 2004 Jul;45(7):2083-90. PubMed PMID: 15223780.

22. Iarossi G, Bertelli M, Maltese PE, Gusson E, Marchini G, Bruson A, et al. Genotype-Phenotype Characterization of Novel Variants in Six Italian Patients with Familial Exudative Vitreoretinopathy. J Ophthalmol. 2017;2017:3080245. PubMed PMID: 28758032; PubMed Central PMCID: PMC5516747.

23. Huang X-Y, Zhuang H, Wu J-H, Li J-K, Hu F-Y, Zheng Y, et al. Targeted next-generation sequencing analysis identifies novel mutations in families with severe familial exudative vitreoretinopathy. Mol Vis. 2017 Aug 23;23:605-613. PubMed PMID: 28867931; PubMed Central PMCID: PMC5568910.

24. Hull S, Arno G, Ostergaard P, Pontikos N, Robson AG, Webster AR, et al. Clinical and Molecular Characterization of Familial Exudative Vitreoretinopathy Associated With Microcephaly. Am J Ophthalmol. 2019 Nov;207:87-98. PubMed PMID: 31077665.

25. Rui Qi, Jinyan Zhu, Xiaoguang Wang, Wenjuan Zhuang, Xunlun Sheng. Analysis of grntype and phenotype of herediatary retinal diseases which are easily misdiagnosed as amblyopia. Chinese Journal of Experimental Ophthalmology. 2019;37(11):888-895.China.

26. Tang M, Ding X, Li J, Hu A, Yuan M, Yang Y, et al. Novel mutations in FZD4 and phenotype-genotype correlation in Chinese patients with familial exudative vitreoretinopathy. Mol Vis. 2016 Jul 30;22:917-32. PubMed PMID: 27555740; PuMed Central PMCID: PMC4968609.

27. Tang M, Sun L, Hu A, Yuan M, Yang Y, Peng X, et al. Mutation Spectrum of the LRP5, NDP, and TSPAN12 Genes in Chinese Patients With Familial Exudative Vitreoretinopathy. Invest Ophthalmol Vis Sci. 2017 Nov 1;58(13):5949-5957. PubMed PMID: 29181528.

28. Drenser KA, Dailey W, Vinekar A, Dalal K, Capone A Jr, Trese MT. Clinical presentation and genetic correlation of patients with mutations affecting the FZD4 gene. Arch Ophthalmol. 2009 Dec;127(12):1649-54. PubMed PMID: 20008721.

29. Carrera W, Ng C, Desler C, Jumper JM, Agarwal A. Novel FZD4 and LRP5 mutations in a small cohort of patients with familial exudative vitreoretinopathy (FEVR). Ophthalmic Genet. 2021 Apr;42(2):200-203. PubMed PMID: 33302760.

30. Jia L-Y, Li X-X, Yu W-Z, Zeng W-t, Liang C. Novel Frizzled-4 Gene Mutations in Chinese Patients With Familial Exudative Vitreoretinopathy. Arch Ophthalmol. 2010 Oct;128(10):1341-9. PubMed PMID: 20938005.

31. Jia L-Y, Ma K. Novel Norrie disease gene mutations in Chinese patients with familial exudative vitreoretinopathy. BMC Ophthalmol. 2021 Feb 15;21(1):84. PubMed PMID: 33588793; PubMed Central PMCID: PMC7885586.

32. Xu Y, Huang L, Li J, Zhang Q, Fei P, Zhu X, et al. Novel mutations in the TSPAN12 gene in Chinese patients with familial exudative vitreoretinopathy. Mol Vis. 2014 Sep 20;20:1296-306. PubMed PMID: 25352738; PubMed Central PMCID: PMC4169774.
