# Supplementary material for: Genotype-phenotype associations in familial exudative vitreoretinopathy: A systematic review and meta-analysis on more than 3200 individuals
Source: PLoS One. 2022 Jul 13;17(7):e0271326. doi: 10.1371/journal.pone.0271326 (PMC9278778; doi:10.1371/journal.pone.0271326)
Supplement: S3 Table — (DOCX) [file pone.0271326.s008.docx]

**S3 Table**. **Probands of degree at different stages of FEVR in different gene groups.**

|  | **Stage** | **LRP5** | **FZD4** | **NDP** | **TSPAN12** |
| --- | --- | --- | --- | --- | --- |
| Probands | Total | 73 | 109 | 42 | 45 |
| Mild | Stage1 | 7（9.59%） | 5（4.59%） | 1（2.38%） | 4（8.89%） |
|  | Stage2 | 10（13.70%） | 21（19.27%） | 5（11.9%） | 11（24.44） |
| Sever | Stage3 | 17（23.29%） | 9（8.26%） | 1（2.38%） | 5（11.11%） |
|  | Stage4 | 23（31.51%） | 47（43.12%） | 5（11.9%） | 19（42.22%） |
|  | Stage5 | 16（21.92%） | 27（24.77%） | 29（69.05%） | 6（13.33%） |
