# Supplementary material for: Genotype-phenotype associations in familial exudative vitreoretinopathy: A systematic review and meta-analysis on more than 3200 individuals
Source: PLoS One. 2022 Jul 13;17(7):e0271326. doi: 10.1371/journal.pone.0271326 (PMC9278778; doi:10.1371/journal.pone.0271326)
Supplement: S4 Table — *When the number of relevant references for a certain gene mutation is less than 3, the statistical results are considered to be no clinical significance and the gene mutation is not counted. AZ = Avascular zone; NV = Neovascularization; Exu = Exudation; ME = Macular ectopia; RLF = Retrolental fibroplasia; RF = Retinal folds; RD = Retinal detachment; TRD = Tractional retinal detachment; Fib = Fibroplasia; CRD = Complete retinal detachment. (DOCX) [file pone.0271326.s009.docx]

**S4 Table. Features of FEVR patients in different gene groups**

| **Gene** | **LRP5** | | **FZD4** | | **NDP** | | **TSPAN12** | |
| --- | --- | --- | --- | --- | --- | --- | --- | --- |
|  | 95%CI | P. Value | 95%CI | P. Value | 95%CI | P. Value | 95%CI | P. Value |
| AZ | 28.1  (10.6-45.5) | <0.01 | 41.2  (22.0-60.3) | <0.01 | 19.3  (5.7-32.9) | <0.01 | 35.7  (8.4-63.1) | <0.01 |
| NV | — | — | 11.1  (2.9-19.2) | <0.01 | — | — | 8.8  (0.0-19.8) | <0.01 |
| Exu | — | — | 7.2  (3.7-10.7) | 0.04 | — | — | — | — |
| Fib | — | — | 0.3  (0.0-1.7) | 0.13 | — | — | — | — |
| ME | — | — | 15.6  (6.1-25.1) | <0.01 | 18.1  (3.7-32.5) | <0.01 | 11.7  (1.6-21.5) | 0.02 |
| RF | 29.5  (14.1-45.0) | <0.01 | 30.1  (24.4-35.8) | 0.06 | 5.5  (0.0-12.9) | 0.04 | 57.3  (30.7-83.8) | <0.01 |
| RLF | 20.9  (1.6-40.2) | <0.01 | 1.3  (0.0-3.3) | 0.53 | 18.0  (1.3-34.8) | <0.01 | 1.6  (0.0-4.4) | 0.51 |
| TRD | — | — | 2.2  (0.0-4.7) | 0.72 | — | — | — | — |
| CRD | 15.6  (10.3-21.0) | 0.28 | 8.8  (5.1-12.6) | 0.04 | 55.7  (27.8-83.7) | <0.01 | 11.8  (6.1-17.4) | 0.19 |
| Total  RD | 51.9  (25.5-78.4) | <0.01 | 32.7  (14.8-50.7) | <0.01 | 64.5  (41.4-87.7) | <0.01 | 29.6  (11.1-48.0) | <0.01 |

*When the number of relevant references for a certain gene mutation is less than 3, the statistical results are considered to be no clinical significance and the gene mutation is not counted.

AZ=Avascular zone; NV=Neovascularization; Exu=Exudation; ME= Macular ectopia; RLF=Retrolental fibroplasia; RF=Retinal folds; RD=Retinal detachment; TRD = Tractional retinal detachment; Fib= Fibroplasia; CRD=Complete retinal detachment;
