# Supplementary material for: Genotype-phenotype associations in familial exudative vitreoretinopathy: A systematic review and meta-analysis on more than 3200 individuals
Source: PLoS One. 2022 Jul 13;17(7):e0271326. doi: 10.1371/journal.pone.0271326 (PMC9278778; doi:10.1371/journal.pone.0271326)
Supplement: S5 Table — *This form contains only those proband who mentioned specific information in the study. OD = Oculus Dexter; OS = Oculus Sinister; OU = Oculus Unati; M = Male; F = Female; NA = No information available; AZ = Avascular zone; NV = Neovascularization; Exu = Exudation; ME = Macular ectopia; RLF = Retrolental fibroplasia; RF = Retinal folds; RD = Retinal detachment; Fib = Fibroplasia; CRD = Complete retinal detachment; TRD = Tractional retinal detachment; ERD = Exudative retinal detachment; PHPV = Persistent Hyperplastic Primary Vitreous; VH = vitreous hemorrhage; EBV = Elongated branching of vessels; IBV = Increased branching of vessels; FRD = Falciform retinal detachment. (DOCX) [file pone.0271326.s010.docx]

**S5 Table. The detailed information and clinical ophthalmological features about each proband in FEVR**

| **No** | **Gender** | **Age** | **Gene** | **Clinical Features** | **Stage** |
| --- | --- | --- | --- | --- | --- |
| 1 | M | 2M | LRP5 | OU: RF, RD | 3(3/3) |
| 2 | F | 2d | LRP5 | OU: AZ | 1(1/1) |
| 3 | F | 2d | LRP5 | OU: AZ | 1(1/1) |
| 4 | F | 1M | LRP5 | OU: CRD | 5(5/5) |
| 5 | M | 4M | LRP5 | OU: CRD | 5(5/5) |
| 6 | M | 13y | LRP5 | OD: AZ, NV; OS: CRD | 5(2/5) |
| 7 | M | 10y | LRP5 | OU: AZ | 1(1/1) |
| 8 | M | 4y | LRP5 | OD: AZ, NV; OS: ME, RD | 3(2/3) |
| 9 | M | 7y | LRP5 | OD: AZ; OS: FRD | 4(1/4) |
| 10 | F | 4y | LRP5 | OD: AZ; OS: FRD | 4(1/4) |
| 11 | F | 4y | LRP5 | OD: AZ, NV; OS: RD, ME | 3(2/3) |
| 12 | M | 1y | LRP5 | OD: AZ, NV, Exu; OS: CRD | 5(2/5) |
| 13 | M | 6y | LRP5 | OD: AZ; OS: CRD | 5(1/5) |
| 14 | F | 2y | LRP5 | OD: FRD; OS: AZ, NV, Exu | 4(4/2) |
| 15 | M | 3y | LRP5 | OU: FRD | 4(4/4) |
| 16 | M | 8y | LRP5 | OD: RD, ME | 3(3/0) |
| 17 | M | 5y | LRP5 | OD: AZ, NV; OS: RD, ME | 3(2/3) |
| 18 | F | 0y | LRP5 | OD: AZ; OS: CRD, Exu | 5(1/5) |
| 19 | F | 6y | LRP5 | OD: AZ; OS: FRD, RLF | 4(1/4) |
| 20 | M | 0y | LRP5 | OU: FRD | 4(4/4) |
| 21 | M | 0y | LRP5 | OU: CRD, Exu | 5(5/5) |
| 22 | M | 1y | LRP5 | OS: CRD | 5(0/5) |
| 23 | M | 0y | LRP5 | OU: AZ, NV, Exu | 2(2/2) |
| 24 | M | 0y | LRP5 | OD: CRD, Cataract; OS: AZ | 5(5/1) |
| 25 | F | 4y | LRP5 | OS: FRD | 4(0/4) |
| 26 | F | 7y | LRP5 | OD: TRD, ME; OS: AZ | 3(3/1) |
| 27 | M | 5y | LRP5 | OD: TRD, ME; OS: AZ | 3(3/1) |
| 28 | M | 3y | LRP5 | OS: CRD | 5(0/5) |
| 29 | M | 4y | LRP5 | OU: AZ, NV | 2(2/2) |
| 30 | M | 1y | LRP5 | OU: FRD | 4(4/4) |
| 31 | F | 4y | LRP5 | OU: AZ, PVT | 1(1/1) |
| 32 | M | 4y | LRP5 | OU: AZ | 1(1/1) |
| 33 | M | 0y | LRP5 | OD: AZ; OS: TRD,ME | 3(1/3) |
| 34 | M | 16y | LRP5 | OU: AZ | 1(1/1) |
| 35 | M | 9y | LRP5 | OS: TRD | 3(0/3) |
| 36 | F | 4y | LRP5 | OD: FRD, RLF; OS: AZ | 4(4/1) |
| 37 | M | 6y | LRP5 | OS: CRD | 5(0/5) |
| 38 | M | 1y | LRP5 | OU: CRD | 5(5/5) |
| 39 | F | 1y | LRP5 | OD: AZ; OS: CRD | 5(1/5) |
| 40 | M | 2y | LRP5 | OD: TRD, MD | 3(3/0) |
| 41 | F | 1y | LRP5 | OD: CRD; OS: FRD | 5(5/4) |
| 42 | M | 2y | LRP5 | OD: FRD; OS: TRD, ME | 4(3/4) |
| 43 | M | 3y | LRP5 | OD: FRD; OS:AZ | 4(4/1) |
| 44 | M | 21y | LRP5 | OU: AZ, NV | 2(2/2) |
| 45 | F | 1y | LRP5 | OD: AZ, NV, Exu; OS: ME, TRD | 3(2/3) |
| 46 | M | 5y | LRP5 | OU: TRD | 3(3/3) |
| 47 | M | 4y | LRP5 | OD: AZ, OS: FRD | 4(1/4) |
| 48 | M | 10y | LRP5 | OD: FRD; OS: AZ | 4(4/1) |
| 49 | M | 2M | LRP5 | OU: RD, RLF, Micro corneal, Flat anterior chamber | 5(5/5) |
| 50 | M | 5M | LRP5 | OU: RLF | 2(2/2) |
| 51 | F | 1y | LRP5 | OD: Optic disc traction; OS: RLF | 2(2/2) |
| 52 | M | 5M | LRP5 | OU: RLF, NYS, Micro corneal | 2(2/2) |
| 53 | F | 6M | LRP5 | OU: RLF | 2(2/2) |
| 54 | M | 9y | LRP5 | OD: RF, TRD, AZ; OS: CRD | 5(4/5) |
| 55 | F | 8y | LRP5 | OU: RF, AZ | 2(2/2) |
| 56 | F | 25y | LRP5 | OS: RD, RF, AZ, FFA leakage, NV | 3(0/3) |
| 57 | M | 19y | LRP5 | OU: RF, RLF, Traction Retinal | 1(1/1) |
| 58 | F | 11M | LRP5 | OU: RF, RD, Traction Retinal | 3(3/3) |
| 59 | F | 41y | LRP5 | OU: Fib | 2(2/2) |
| 60 | M | 2M | FZD4 | OU: CRD | 5(5/5) |
| 61 | F | 3M | FZD4 | OU: FRD | 4(4/4) |
| 62 | F | 9y | FZD4 | OU: AZ, NV | 2(2/2) |
| 63 | M | 2y | FZD4 | OD: AZ; OS: FRD,RLF | 4(1/4) |
| 64 | F | 6y | FZD4 | OU: CRD | 5(5/5) |
| 65 | M | 2y | FZD4 | OD: CRD | 5(5/0) |
| 66 | M | 0y | FZD4 | OU: NV | 2(2/2) |
| 67 | M | 8y | FZD4 | OD: AZ; OS: CRD | 5(1/5) |
| 68 | M | 1y | FZD4 | OU: FRD | 4(4/4) |
| 69 | M | 4y | FZD4 | OD: AZ; OS: CRD | 5(1/5) |
| 70 | M | 7y | FZD4 | OU: FRD | 4(4/4) |
| 71 | F | 3y | FZD4 | OU: FRD | 4(4/4) |
| 72 | M | 1y | FZD4 | OD: AZ, NV; OS: FRD | 4(2/4) |
| 73 | M | 1y | FZD4 | OS: FRD | 4(0/4) |
| 74 | M | 4y | FZD4 | OS: FRD | 4(0/4) |
| 75 | M | 11y | FZD4 | OD: AZ, NV | 2(2/0) |
| 76 | M | 9y | FZD4 | OD: TRD, Exu; OS: FRD | 4(3/4) |
| 77 | M | 0y | FZD4 | OU: AZ, NV | 2(2/2) |
| 78 | M | 6y | FZD4 | OD: FRD | 4(4/0) |
| 79 | M | 15y | FZD4 | OU: CRD | 5(5/5) |
| 80 | M | 2y | FZD4 | OD: FRD | 4(4/0) |
| 81 | M | 3y | FZD4 | OU: AZ, NV | 2(2/2) |
| 82 | M | 2y | FZD4 | OD: AZ; OS: CRD | 5(1/5) |
| 83 | M | 2y | FZD4 | OD: CRD | 5(5/0) |
| 84 | M | 1y | FZD4 | OD: AZ; OS: FRD, Exu | 4(1/4) |
| 85 | M | 1y | FZD4 | OD: FRD; OS: TRD, ME | 4(4/3) |
| 86 | M | 6y | FZD4 | OU: FRD | 4(4/4) |
| 87 | M | 0y | FZD4 | OD: NV; OS: TRD | 3(2/3) |
| 88 | M | 1y | FZD4 | OD: Exu, NV; OS: Exu, CRD | 5(2/5) |
| 89 | F | 5y | FZD4 | OD: FRD; OS: AZ | 4(4/1) |
| 90 | M | 5y | FZD4 | OD: CRD; OS: FRD | 5(5/4) |
| 91 | M | 0y | FZD4 | OD: AZ, NV; OS: CRD | 5(2/5) |
| 92 | M | 3y | FZD4 | OU: CRD | 5(5/5) |
| 93 | F | 1y | FZD4 | OD: FRD; OS: NV | 4(4/2) |
| 94 | F | 25y | FZD4 | OU: TRD, ME | 3(3/3) |
| 95 | M | 4y | FZD4 | OD: CRD | 5(5/0) |
| 96 | F | 2y | FZD4 | OD: AZ; OS: CRD | 5(1/5) |
| 97 | M | 2y | FZD4 | OD: CRD | 5(5/0) |
| 98 | F | 0y | FZD4 | OD: CRD; OS: FRD | 5(5/4) |
| 99 | M | 6y | FZD4 | OU: AZ, NV | 2(2/2) |
| 100 | M | 9y | FZD4 | OD: AZ; OS: TRD, ME | 3(1/3) |
| 101 | M | 8y | FZD4 | OU: FRD | 4(4/4) |
| 102 | F | 9y | FZD4 | OD: FRD; OS: AZ | 4(4/1) |
| 103 | F | 20y | FZD4 | OD: IBV; OS: AZ, RD, NV, Fib | 3(0/3) |
| 104 | M | 5y | FZD4 | OD: IBV; OS: Optic disc traction | 2(0/2) |
| 105 | F | 4y | FZD4 | OU: Optic disc traction; OS: Fib | 2(2/2) |
| 106 | F | 4y | FZD4 | OD: EBV, OS: Optic disc traction, Fib | 2(1/2) |
| 107 | M | 9y | FZD4 | OD: RLF, Lens ectopia; OS: AZ, Brush for vessels | 2(2/2) |
| 108 | F | 2y | FZD4 | OD: Optic disc traction, OS: RF | 4(2/4) |
| 109 | M | 9y | FZD4 | OD: AZ; OS: RF | 3(1/3) |
| 110 | M | 2y | FZD4 | OD: RF; OS: AZ | 3(3/2) |
| 111 | M | 13y | FZD4 | OU: ME, Exu, FFA leakage, macular edema, Posterior vitreous traction, Esotropia | 4(4/4) |
| 112 | M | 4y | FZD4 | OU: FRD | 4(4/4) |
| 113 | F | 14y | FZD4 | OD: MD, AZ, NV, Exu; OS: TRD | 5(2/5) |
| 114 | M | 11y | FZD4 | OD: TRD; OS: AZ, ME, IBV, EBV | 5(5/2) |
| 115 | M | 8y | FZD4 | OU: AZ, IBV, EBV; OS: ME | 1(1/1) |
| 116 | F | 13y | FZD4 | OD: AZ, NV, IBV, EBV, Exu; OS: FRD | 4(4/2) |
| 117 | M | 13y | FZD4 | OD: AZ, NV, IBV, EBV, Exu; OS: FRD | 4(2/4) |
| 118 | M | 8y | FZD4 | OU: FRD | 4(4/4) |
| 119 | M | 15y | FZD4 | OU: AZ, IBV; OD: ME | 1(1/1) |
| 120 | M | 12y | FZD4 | OD: FRD; OS: AZ, IBV, NV, Exu | 4(4/2) |
| 121 | M | 6y | FZD4 | OD: FRD; OS: ME, AZ, NV, Exu, IBV, EBV | 4(4/2) |
| 122 | F | 3y | FZD4 | OD: FRD; OS: MD, AZ, IBV, EBV | 4(4/1) |
| 123 | M | 5y | FZD4 | OU: AZ, IBV, EBV; OS: ME, NV, Exu | 2(1/2) |
| 124 | F | 7y | FZD4 | OD: FRD; OS: AZ, IBV, EBV | 4(4/1) |
| 125 | M | 10y | FZD4 | OU: AZ; OD: ME; OS: IBV, EBV | 1(1/1) |
| 126 | F | 2M | FZD4 | OU: FRD | 4(4/4) |
| 127 | M | 2y | FZD4 | OU: FRD | 4(4/4) |
| 128 | M | 4y | FZD4 | OD: AZ, IBV, EBV; OS: FRD | 4(1/4) |
| 129 | M | 6M | FZD4 | OD:AZ, EBV; OS: FRD | 4(1/4) |
| 130 | F | 26y | FZD4 | OU: AZ, IBV, EBV, NV; OD: Exu, VH | 2(2/2) |
| 131 | F | 15y | FZD4 | OD: FRD; OS: AZ, NV, IBV, EBV, Exu, VH | 4(4/1) |
| 132 | M | 10y | FZD4 | OU: IBV, EBV, NV, AZ; OD: ME, Exu | 2(2/2) |
| 133 | F | 1y | FZD4 | OU: AZ | 1(1/1) |
| 134 | M | 2y | FZD4 | OU: AZ | 1(1/1) |
| 135 | M | 18y | FZD4 | OU: AZ, Retinal Hole; OD: ME | 2(2/1) |
| 136 | M | 13y | FZD4 | OU: AZ, NV; OS: ME, RD, Fib, Retinal Hole; OS: VH | 4(2/4) |
| 137 | M | 53y | FZD4 | OU: AZ, ME; OS: NV; OS: Fib | 2(2/2) |
| 138 | F | 15y | FZD4 | OU: AZ, ME, RD | 4(4/4) |
| 139 | F | 8M | FZD4 | OU: RD, RLF, NYS | 5(5/5) |
| 140 | M | 8M | FZD4 | OU: RF, PHPV | 2(2/2) |
| 141 | F | 4y | FZD4 | OU: AZ, ME | 1(1/1) |
| 142 | F | 3y | FZD4 | OU: RF, ME, RD | 4(4/4) |
| 143 | F | 18y | FZD4 | OU: AZ, Exu | 1(1/1) |
| 144 | M | 9y | FZD4 | OU: AZ, NV; OD: Fib | 2(2/2) |
| 145 | M | 6M | FZD4 | OU: Exu; OD: ME; OS: FRD | 4(2/4) |
| 146 | F | 8M | FZD4 | OU: RF, PHPV | 2(2/2) |
| 147 | F | 1y | FZD4 | OU: Optic disc traction | 2(2/2) |
| 148 | F | 4y | FZD4 | OU: ME, AZ; OS: Fib | 2(2/2) |
| 149 | F | 5y | FZD4 | OU: RF, ME, RD | 4(4/4) |
| 150 | M | 2y | FZD4 | OU: ME, RD | 3(3/3) |
| 151 | F | 35y | FZD4 | OD: RF, Vitreoretinal traction, Optic disc traction; OS: FRD | 4(2/4) |
| 152 | M | 6M | FZD4 | OD: CRD; OS: RF, Optic disc traction, Vitreoretinal traction | 5(5/2) |
| 153 | M | 4y | FZD4 | OU: AZ, Exu, Vitreous Opacity; OD: ME; OS: FRD | 4(2/4) |
| 154 | M | 18y | FZD4 | OU: Vitreous Opacity; OD: ME, AZ, FRD, Exu, Retinal Hole | 5(4/5) |
| 155 | F | 21y | FZD4 | OU: AZ, Exu; OD: ERD; OS: Retinal Hole | 5(5/1) |
| 156 | M | 1y | FZD4 | OU: AZ, ME, FRD; OD: Vitreous Opacity | 4(4/4) |
| 157 | F | 1y | FZD4 | OS: FRD, AZ, ME, Vitreous Opacity | 5(5/4) |
| 158 | M | 6M | FZD4 | OD: NV; OS: Corneal Opacity, RLF, CRD | 5(2/5) |
| 159 | M | 57y | FZD4 | OU: RF, OS: RD | 3(1/3) |
| 160 | F | 30y | FZD4 | OU: NV, Traction Retinal | 2(2/2) |
| 161 | M | 3y | FZD4 | OS: RF, Traction Retinal, NV, RD | 3(0/3) |
| 162 | M | 1M | NDP | OU: CRD | 5(5/5) |
| 163 | M | 2M | NDP | OU: CRD | 5(5/5) |
| 164 | M | 1M | NDP | OU: CRD | 5(5/5) |
| 165 | M | 0y | NDP | OU: CRD | 5(5/5) |
| 166 | M | 0y | NDP | OU: CRD, Cataract | 5(5/5) |
| 167 | M | 3y | NDP | OU: CRD; OS: Cataract | 5(5/5) |
| 168 | M | 0y | NDP | OU: CRD; OS: RLF | 5(5/5) |
| 169 | M | 1y | NDP | OU: CRD; OD: RLF; OS: Cataract | 5(5/5) |
| 170 | M | 2y | NDP | OD: CRD | 5(5/0) |
| 171 | M | 2y | NDP | OU: CRD | 5(5/5) |
| 172 | M | 0y | NDP | OU: CRD, Cataract | 5(5/5) |
| 173 | M | 11y | NDP | OD: CRD; OS: AZ | 5(5/1) |
| 174 | M | 1y | NDP | OU: CRD; OD: RLF; OS: Cataract | 5(5/5) |
| 175 | M | 0y | NDP | OD: CRD; OS: FRD | 5(5/4) |
| 176 | M | 12y | NDP | OU: CRD | 5(5/5) |
| 177 | M | 0y | NDP | OU: CRD, Cataract | 5(5/5) |
| 178 | M | 15y | NDP | OD: CRD; OS: FRD | 5(5/4) |
| 179 | M | 2y | NDP | OD: CRD | 5(5/0) |
| 180 | M | 11y | NDP | NA | NA |
| 181 | M | 7y | NDP | OD: AZ, FFA leakage; OS: RF, RD, NYS, Micro corneal | 3(2/3) |
| 182 | M | 4M | NDP | OD: RD, Exu, FFA leakage; OS: AZ, Exu | 4(4/1) |
| 183 | M | 3M | NDP | OD: RLF; OS: CRD, NYS, Optic disc traction | 5(5/5) |
| 184 | M | 4M | NDP | OU: RF | 5(4/5) |
| 185 | M | Birth | NDP | OU: CRD, RLF | 5(5/5) |
| 186 | M | 6y | NDP | OU: ME, RF | 4(4/4) |
| 187 | F | 1y | NDP | OU: ME, RF, Esotropia | 4(4/4) |
| 188 | M | 5M | NDP | OD: AZ; OS: MD, Optic disc traction | 5(3/5) |
| 189 | M | 3M | NDP | OU: CRD, RLF | 5(5/5) |
| 190 | M | 24y | NDP | OU: AZ, EBV, FFA leakage | 2(2/2) |
| 191 | M | 3M | NDP | OU: CRD, RLF, Corneal Opacity | 5(5/5) |
| 192 | M | 3M | NDP | OU: CRD, Flat anterior chamber, RLF, Corneal Opacity | 5(5/5) |
| 193 | M | 6M | NDP | OD: RLF, CRD, Flat anterior chamber; OS: AZ, NV | 5(5/2) |
| 194 | M | 6y | NDP | OU: AZ, ME, PHPV, Fib | 2(2/2) |
| 195 | M | 5y | NDP | OU: AZ, Fib, PHPV | 2(2/2) |
| 196 | M | 21y | NDP | OU: AZ, ME, Fib | 2(2/2) |
| 197 | M | 1y | NDP | OU: CRD, RLF, Flat anterior chamber, Corneal Opacity | 5(5/5) |
| 198 | M | 1y | NDP | OU: CRD, RLF, Flat anterior chamber | 5(5/5) |
| 199 | M | 11M | NDP | OU: AZ, ME | 4(4/4) |
| 200 | M | 4M | NDP | OD: RD, AZ; OS:ME | 5(5/4) |
| 201 | M | 6M | NDP | OU: ME | 4(4/4) |
| 202 | M | 2y | NDP | OU: AZ, ME | 4(4/4) |
| 203 | M | 1M | TSPAN12 | OU: RF, OS: CRD | 5(4/5) |
| 204 | M | 5y | TSPAN12 | OU: AZ | 2(2/2) |
| 205 | M | 3y | TSPAN12 | OU: AZ, NV, Exu | 2(2/2) |
| 206 | M | 4y | TSPAN12 | OU: AZ | 2(2/2) |
| 207 | M | 7y | TSPAN12 | OU: AZ, NV | 4(4/4) |
| 208 | F | 3y | TSPAN12 | OD: TRD, ME; OS: FRD, RLF | 4(3/4) |
| 209 | F | 5y | TSPAN12 | OU: AZ | 2(2/1) |
| 210 | M | 10y | TSPAN12 | OU: AZ; OD: NV | 4(4/4) |
| 211 | M | 4y | TSPAN12 | OU: FRD | 4(4/4) |
| 212 | M | 1y | TSPAN12 | OU: AZ, OS: NV | 2(1/2) |
| 213 | M | 1y | TSPAN12 | OU: AZ | 4(4/4) |
| 214 | M | 4y | TSPAN12 | OU: AZ, NV, Exu | 2(2/2) |
| 215 | M | 3y | TSPAN12 | OU: AZ | 4(4/4) |
| 216 | F | 4y | TSPAN12 | OU: ME, NV, PVT | 2(2/2) |
| 217 | M | 2y | TSPAN12 | OU: AZ | 1(1/1) |
| 218 | M | 1y | TSPAN12 | OD: FRD; OS: TRD, ME | 4(4/3) |
| 219 | M | 6y | TSPAN12 | OU: AZ, NV | 4(4/4) |
| 220 | M | 1y | TSPAN12 | OU: AZ | 4(4/3) |
| 221 | M | 1y | TSPAN12 | OU: FRD, RLF | 4(4/4) |
| 222 | M | 5y | TSPAN12 | OD: AZ, NV, Exu; OS: FRD | 4(2/4) |
| 223 | F | 0y | TSPAN12 | OU: AZ, NV | 4(4/4) |
| 224 | M | 5y | TSPAN12 | OU: AZ, OS: NV | 2(1/2) |
| 225 | M | 6y | TSPAN12 | OU: AZ | 1(1/1) |
| 226 | F | 2y | TSPAN12 | OU: AZ, NV | 3(3/3) |
| 227 | F | 14y | TSPAN12 | OU: AZ | 1(1/1) |
| 228 | M | 23y | TSPAN12 | OD: AZ, OS: FRD | 5(5/4) |
| 229 | M | 1y | TSPAN12 | OD: AZ, NV; OS: FRD | 4(3/4) |
| 230 | F | 6y | TSPAN12 | OU:AZ | 4(4/4) |
| 231 | M | 1y | TSPAN12 | OU: RF | 3(3/3) |
| 232 | F | 34y | TSPAN12 | OU: AZ, FFA leakage; OS: NV | 2(2/2) |
| 233 | F | 34y | TSPAN12 | OU: AZ, FFA leakage, NV; OS: RD, RF | 3(1/3) |
| 234 | F | 15y | TSPAN12 | OU: AZ; OD: FRD, RLF | 4(4/2) |
| 235 | M | 11y | TSPAN12 | OU: FRD, Fib; OS: AZ | 4(4/4) |
| 236 | F | 12y | TSPAN12 | OU: AZ, ME, Fib | 2(2/2) |
| 237 | F | 5M | TSPAN12 | OD: Fib; OS: RF | 2(2/2) |
| 238 | M | 4y | TSPAN12 | OU: IBV, FFA leakage; OD: Traction Retinal; OS: EBV | 2(2/2) |
| 239 | F | 3y | TSPAN12 | OU: RF | 2(2/2) |
| 240 | M | 0y | TSPAN12 | OU: AZ, IBV, FFA leakage; OS: RF | 2(0/2) |
| 241 | M | 4M | TSPAN12 | OU: RF | 2(2/2) |
| 242 | M | 1y | TSPAN12 | OU: RF | 2(2/2) |
| 243 | M | 9y | TSPAN12 | OU: AZ, IBV, FFA leakage; OS: Fib | 2(1/2) |
| 244 | F | 1y | TSPAN12 | OD: RD; OS: RF | 5(5/2) |
| 245 | M | 1y | TSPAN12 | OD: NV; OS: RF | 2(2/2) |
| 246 | M | 6M | TSPAN12 | OU: RF | 2(2/2) |
| 247 | M | 0y | TSPAN12 | OU: IBV; OD: Traction Retinal, Fib | 2(2/1) |
| 248 | M | 0y | TSPAN12 | OU: AZ; OD: Traction Retinal; OS: IBV, FFA leakage | 1(1/1) |
| 249 | M | 6y | TSPAN12 | OS: Traction Retinal | 2(0/2) |
| 250 | M | 12y | TSPAN12 | OD: AZ, Fib, IBV, FFA leakage; OS: RD, EBV | 5(2/5) |
| 251 | M | 14y | TSPAN12 | OU: RD, FFA leakage; OD: IBV; OS: EBV, NV | 5(5/5) |
| 252 | M | 4M | TSPAN12 | OU: RF, AZ; OD: Retinal pigment epithelium dystrophy | 2(2/2) |
| 253 | F | 2y | TSPAN12 | OD: RF, OS: Traction Retinal | 2(2/2) |
| 254 | F | 3M | TSPAN12 | OD: AZ; OS: RF | 2(1/2) |
| 255 | F | 35y | TSPAN12 | OU: Fib, Traction Retinal ,FFA leakage; OS: ME | 2(2/2) |
| 256 | M | 3y | TSPAN12 | OU: AZ, Fib; OD: IBV; OS: EBV | 2(2/2) |
| 257 | M | 1y | TSPAN12 | OU: Traction Retinal; OS: Fib | 2(2/2) |
| 258 | M | 1y | TSPAN12 | OU: RF, ME | 4(4/4) |
| 259 | F | 2y | TSPAN12 | OU: RF | 4(4/4) |
| 260 | M | 5y | TSPAN12 | OU: RF, ME, RD | 2(2/2) |

*This form contains only those proband who mentioned specific information in the study.

OD=Oculus Dexter; OS=Oculus Sinister; OU=Oculus Unati; M=Male; F=Female; NA=No information available; AZ=Avascular zone; NV=Neovascularization; Exu=Exudation; ME= Macular ectopia; RLF=Retrolental fibroplasia; RF=Retinal folds; RD=Retinal detachment; Fib= Fibroplasia; CRD=Complete retinal detachment; TRD = Tractional retinal detachment; ERD=Exudative retinal detachment; PHPV= Persistent Hyperplastic Primary Vitreous; VH= vitreous hemorrhage; EBV= Elongated branching of vessels; IBV=Increased branching of vessels; FRD= Falciform retinal detachment
