# Supplementary material for: Genotype-phenotype associations in familial exudative vitreoretinopathy: A systematic review and meta-analysis on more than 3200 individuals
Source: PLoS One. 2022 Jul 13;17(7):e0271326. doi: 10.1371/journal.pone.0271326 (PMC9278778; doi:10.1371/journal.pone.0271326)
Supplement: S1 File — (DOC) [file pone.0271326.s011.doc]

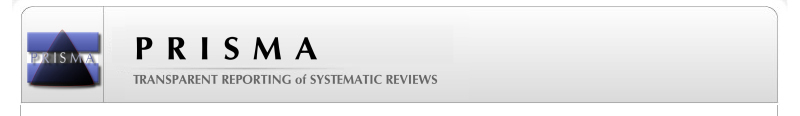
**PRISMA 2009 Flow Diagram**

**Screening**

**Included**

**Eligibility**

**Identification**

Records identified through database searching
(n =970 )

Additional records identified through other sources
(n = 0 )

Records after duplicates removed
(n =407 )

Records screened
(n =563 )

Records excluded
(n =475 )

Full-text articles assessed for eligibility
(n = 88 )

Full-text articles excluded, with reasons
(n = 56 )

Studies included in qualitative synthesis
(n =32 )

Studies included in quantitative synthesis (meta-analysis)
(n =32 )
